# Computational modeling establishes mechanotransduction as a potent modulator of the mammalian circadian clock

**DOI:** 10.1242/jcs.261782

**Published:** 2024-09-09

**Authors:** Emmet A. Francis, Padmini Rangamani

**Affiliations:** ^1^ Department of Mechanical and Aerospace Engineering, University of California, San Diego, La Jolla, CA 92093, USA; ^2^ Department of Pharmacology, School of Medicine, University of California, San Diego, La Jolla, CA 92093, USA

**Keywords:** MRTF, YAP/TAZ, Circadian clock, Mechanotransduction, Systems biophysics

## Abstract

Mechanotransduction, which is the integration of mechanical signals from the external environment of a cell to changes in intracellular signaling, governs many cellular functions. Recent studies have shown that the mechanical state of the cell is also coupled to the cellular circadian clock. To investigate possible interactions between circadian rhythms and cellular mechanotransduction, we have developed a computational model that integrates the two pathways. We postulated that translocation of the transcriptional regulators MRTF (herein referring to both MRTF-A and MRTF-B), YAP and TAZ (also known as YAP1 and WWTR1, respectively; collectively denoted YAP/TAZ) into the nucleus leads to altered expression of circadian proteins. Simulations from our model predict that lower levels of cytoskeletal activity are associated with longer circadian oscillation periods and higher oscillation amplitudes, which is consistent with recent experimental observations. Furthermore, accumulation of YAP/TAZ and MRTF in the nucleus causes circadian oscillations to decay in our model. These effects hold both at the single-cell level and within a population-level framework. Finally, we investigated the effects of mutations in YAP or lamin A, the latter of which result in a class of diseases known as laminopathies. *In silico*, oscillations in circadian proteins are substantially weaker in populations of cells with mutations in YAP or lamin A, suggesting that defects in mechanotransduction can disrupt the circadian clock in certain disease states; however, reducing substrate stiffness in the model restores normal oscillatory behavior, suggesting a possible compensatory mechanism. Thus, our study identifies that mechanotransduction could be a potent modulatory cue for cellular clocks and that this crosstalk can be leveraged to rescue the circadian clock in disease states.

## Teaser

**Highlighted Article:** A mathematical model connecting mechanotransduction and circadian clocks shows that mechanical cues can alter the frequency, amplitude and stability of intrinsic circadian oscillations in mammalian cells.

## INTRODUCTION

The 24 h cycles known as circadian rhythms are a hallmark of life on earth. In mammals, organism-wide circadian oscillations are regulated by signals from the central circadian clock, which is located in the suprachiasmatic nucleus of the hypothalamus (Welsh et al., 2010; Evans, 2016). This central clock consists of a population of cells that exhibit oscillations in the expression of circadian proteins, including brain and muscle ARNT-like protein 1 (BMAL1), period proteins (PER1 and PER2, referred to collectively here as PER), cryptochromes (CRY1 and CRY2, referred to collectively here as CRY) and nuclear receptor family subfamily 1 group D member 1 (NR1D1), commonly referred to as REV-ERBα (Takahashi, 2017; Buhr and Takahashi, 2013). These oscillations are synchronized by environmental cues such as light–dark cycles via the process of entrainment. Remarkably, cells from peripheral tissues also exhibit 24 h patterns of protein expression in these same circadian proteins even when removed from the body, demonstrating the intrinsic nature of the cellular circadian clock. Furthermore, these circadian cycles influence a wide range of functions in cells; for instance, ∼43% of protein-coding genes in mice have been shown to undergo 24 h cycles in transcription somewhere in the body (Zhang et al., 2014). Recent experimental studies have revealed connections between disease states and disrupted circadian rhythms in different cell types (Shafi and Knudsen, 2019; Mason et al., 2020).

Mechanical and chemical cues within a tissue may also serve as circadian clock regulators (Streuli and Meng, 2019). Although it has long been known that a serum shock synchronizes the circadian oscillations of cells in culture (Balsalobre et al., 1998), it has recently been found that cytoskeletal activity also contributes to clock regulation (Gerber et al., 2013; Esnault et al., 2014). Additional studies have found that the circadian clock is mechanosensitive – the strength of oscillations changes depending on substrate stiffness (Yang et al., 2017; Williams et al., 2018). Xiong et al. have recently analyzed this phenomenon in detail, demonstrating that altering the mechanical state of fibroblasts or U2OS cells by either treating with actomyosin inhibitors or seeding cells on substrates of different stiffness leads to distinct changes in the period and amplitude of circadian oscillations (Xiong et al., 2022). In particular, the authors found that decreases in cytoskeletal activity are generally associated with increases in PER2 oscillation period and amplitude. This was shown to depend on nuclear activation of serum response factor (SRF) by myocardin-related transcription factor (MRTF, herein referring to both MRTF-A, also known as MAL, and MRTF-B). In a different study, Abenza et al. have found that increased nuclear levels of yes-associated protein (YAP, also known as YAP1) and transcriptional coactivator with PDZ-binding motif (TAZ, also known as WWTR1), which are collectively termed YAP/TAZ, correlate with significant perturbations to circadian oscillations in fibroblasts (Abenza et al., 2023), and this effect is at least partially mediated by transcriptional enhanced associate domain proteins (referred to collectively here as TEADs). Taken together, these findings suggest that the cell circadian clock is affected by the key players in cellular mechanotransduction, namely MRTF and YAP/TAZ.

The nuclear translocation of transcriptional regulators such as YAP/TAZ and MRTF is a critical downstream event in cellular mechanotransduction, the process by which cells respond to their mechanical environment via force-dependent changes in biochemical signaling networks. For instance, cells on stiffer substrates show increased levels of phosphorylation of focal adhesion kinase (here FAK, also known as PTK2), leading to downstream changes in cytoskeletal activity and nuclear localization of YAP/TAZ and MRTF. YAP/TAZ and MRTF then bind transcription factors such as TEADs and SRF, causing changes in gene expression. Accordingly, tissue stiffness is a crucial determinant of cell behavior, controlling processes from stem cell differentiation to cell migration (Janmey et al., 2020). Additionally, changes to tissue stiffness are often observed in diseases such as cancers, likely contributing to their pathophysiologies (Chin et al., 2016). Here, we examine how changes in tissue stiffness or other mechanical factors might disrupt the circadian clock, perhaps contributing to certain cases of disease progression.

How might cell signaling due to mechanotransduction and circadian oscillations be connected? In this study, we sought to answer this question by using computational modeling. Building on the rich history of modeling circadian oscillations in single cells (Asgari-Targhi and Klerman, 2019; Leloup and Goldbeter, 2003; Lema et al., 2000) and independent models of stiffness-dependent mechanotransduction (Sun et al., 2016; Scott et al., 2021), we developed a model of mechanotransduction-induced perturbations to the cell circadian clock. We constrained our model to recently published experiments, and then used it to investigate the coupling between cell signaling and circadian oscillations in single cells and populations of cells. Finally, we investigated how mutations in YAP or lamin A might impact circadian oscillations. We found that such mutations can significantly weaken circadian oscillations, but this effect can be counteracted by reductions in substrate stiffness. Our model has implications for laminopathies, a class of diseases marked by mutations in the gene encoding lamin A (*LMNA*), as well as for diseases marked by changes in local tissue stiffness.

## RESULTS

### Model development

**Fig. 1.**
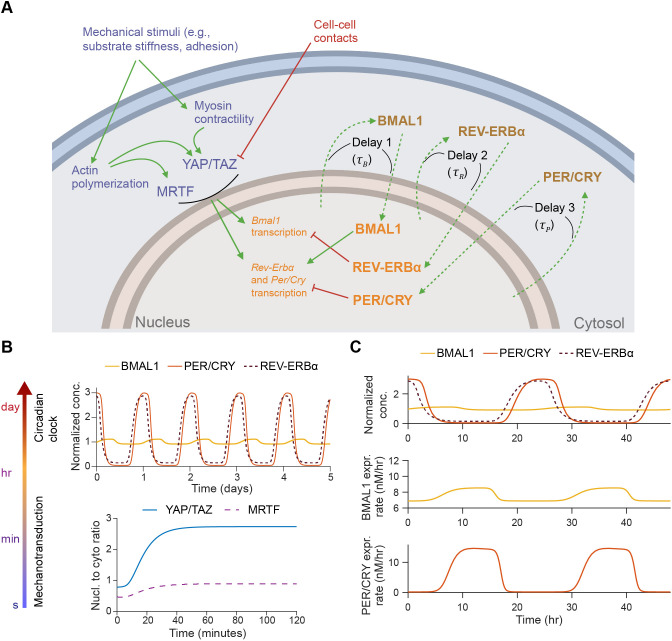
**A coupled model of mechanotransduction and the circadian clock.** (A) Schematic of pathways for YAP/TAZ and MRTF-mediated mechanotransduction and the mammalian circadian clock. Mechanical stimuli such as substrate stiffness and cell–substrate adhesion induce changes in cytoskeletal activity that lead to nuclear translocation of YAP/TAZ and MRTF. Cell–cell contacts also inhibit YAP/TAZ activation via a LATS-dependent pathway. Nuclear YAP/TAZ and MRTF are posited to induce changes in the expression of BMAL1, PER, CRY and REV-ERBα proteins, altering circadian oscillations. In parallel, increases in nuclear BMAL1 enhance transcription of genes encoding PER, CRY and REV-ERBα, while increases in nuclear REV-ERBα inhibit transcription of *Bmal1* and increases in nuclear PER/CRY inhibit transcription of genes encoding PER, CRY and REV-ERBα. Dashed lines indicate delays associated with transcription, translation, post-translational modifications and nuclear translocation of BMAL1, PER/CRY and REV-ERBα. The schematic was created with Biorender.com. (B) Separation of timescales from mechanotransduction pathways to circadian oscillations. Events downstream of mechanical stimuli lead to changes in YAP/TAZ and MRTF nuclear concentrations within tens of minutes (lower plot), whereas changes in the concentration of circadian proteins extend over hours to days (upper plot). (C) Simulated dynamics of single-cell circadian oscillations. BMAL1, PER/CRY and REV-ERBα concentrations all oscillate with periods close to 1 day, as shown in the upper plot. Rates of expression of BMAL1 and PER/CRY are depicted in the lower plots. Conc., concentration; cyto, cytosolic; expr., expression; nucl., nuclear.

We developed a mathematical model that includes YAP/TAZ- and MRTF-mediated mechanotransduction as well as circadian oscillations in the expression of BMAL1, PER and CRY (collectively referred to as PER/CRY), and REV-ERBα (Fig. 1A). In the model, the process of mechanosensing leading to nuclear translocation of YAP/TAZ and/or MRTF occurs at a faster timescale compared to changes in the expression of circadian proteins (Fig. 1B). Assuming that YAP/TAZ and MRTF levels remain relatively constant over several days, we compute their steady-state nuclear concentrations and use these as inputs to the dynamical model of circadian oscillations. Furthermore, in all cases, we assume that species are well-mixed within a given cellular compartment (plasma membrane, cytosol, nuclear membrane or interior of the nucleus), allowing us to treat these systems using ordinary differential equations (ODEs) and delay differential equations (DDEs). The mechanotransduction model includes chemical species in all four compartments, whereas the three circadian species are all within the nucleus. We briefly summarize the mechanotransduction and circadian clock modules of our model below.

### YAP/TAZ and MRTF mechanotransduction model

Our mechanotransduction model is built from the model originally developed by Sun et al. (2016) and then expanded by Scott et al. (2021). In brief, cell–substrate adhesion leads to substrate-stiffness-dependent phosphorylation of FAK adjacent to the cell membrane, triggering downstream activation of the GTPase RhoA. RhoA then activates both Rho kinases (ROCK1 and ROCK2, collectively termed ROCK) and mDia-family formins, which promote myosin activity and actin polymerization, respectively. ROCK also indirectly promotes actin polymerization by activating LIM kinases (LIMKs), which inactivate F-actin-severing cofilin proteins. Upon activation, myosin and actin form stress fibers, leading to the dephosphorylation of YAP and TAZ in the cytosol and their translocation into the nucleus. When simulating different cell densities, we also assume that cell–cell contacts induce increased phosphorylation of YAP/TAZ via a pathway that is dependent on LATS1 and LATS2 kinases (collectively termed LATS), causing more YAP/TAZ to remain cytosolic. Actin polymerization also permits the release of G-actin-sequestered MRTF, which can then translocate into the nucleus. As in the work of Scott et al. (2021), we assume that the opening of nuclear pore complexes (NPCs) depends on the phosphorylation state of lamin A; in particular, lamin A is increasingly dephosphorylated on stiffer substrates, leading to its incorporation into the nuclear lamina and concomitant stretching of NPCs (Swift et al., 2013). This allows for increased transport of both YAP/TAZ and MRTF into the nucleus. The model of Scott et al. does not include MRTF (Scott et al., 2021), whereas the work of Sun et al. does not consider contributions of lamin A and the NPC to the nuclear transport of MRTF and YAP/TAZ (Sun et al., 2016). Here, we combined elements of both models to fully examine both YAP/TAZ and MRTF nuclear translocation. This mechanotransduction model includes 25 species, which can be reduced to 13 ODEs by accounting for mass conservation. We solved for steady-state values by setting each differential equation equal to zero; the resulting expressions for each steady-state quantity are given in Table S2. For all YAP/TAZ-related parameters, we used the values previously estimated for mammalian cells (Scott et al., 2021) (see Table S3), and MRTF-specific parameters and sensitivities to different treatment conditions were calibrated as described below. Baseline values for MRTF rate constants were set to match the values used for YAP/TAZ where applicable (Table S4).

### Circadian clock model

There is a vast body of literature on modeling cell circadian oscillations (reviewed in Asgari-Targhi and Klerman, 2019). Here, rather than attempt to recapitulate the mammalian circadian clock in full mechanistic detail, we developed a reduced-order model, including the dynamics of positive and negative clock regulators through three representative species, *B* (nuclear concentration of BMAL1), *P* (nuclear concentration of PER and CRY proteins) and *R* (nuclear concentration of REV-ERBα). This reduced approach was made possible by using DDEs, as in multiple previous circadian models of varying complexity (Lema et al., 2000; Sriram et al., 2006; Smolen et al., 2001). In addition to these DDE models, we integrated knowledge from previous ODE-based models of the mammalian circadian clock (Leloup and Goldbeter, 2003; Mirsky et al., 2009; Forger and Peskin, 2003).

In our model, BMAL1 induces the expression of REV-ERBα and PER/CRY after translocating into the nucleus and forming a complex with CLOCK (circadian locomotor output cycles kaput). CLOCK levels remain relatively constant over time (Shearman et al., 2000), and so it is not treated as a separate dynamical variable here. Upon its expression and transport into the nucleus, REV-ERBα inhibits the expression of BMAL1 (Takahashi, 2017). This effect is modeled as an inhibition with delay *τ*_*B*_, which represents the time between changes in transcription and associated changes in the nuclear concentration of BMAL1. This includes the time for transcription, translation, post-translational modifications and shuttling of BMAL1 into the nucleus. As a starting estimate for this parameter, we used the time gap between peak BMAL1 transcription and peak nuclear BMAL1, which has been measured at 12–16 h in mouse fibroblasts (Tamaru et al., 2003). Writing *R*[*t*−*τ*_*B*_] as the nuclear concentration of REV-ERBα at time *t*−*τ*_*B*_, the REV-ERBα-regulated BMAL1 expression level is:
(1)

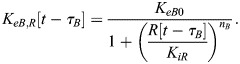


Similarly, writing the delay between changes in PER/CRY (*P*) transcription and eventual changes in nuclear PER/CRY as *τ*_*P*_ [approximated to be 6–9 h based on measurements in mouse cells (Yagita et al., 2001, 2002; Lee et al., 2001)], the BMAL1-regulated expression of PER/CRY is:
(2)

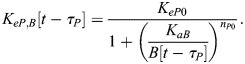


PER/CRY, in turn, binds to the BMAL1–CLOCK complex in the nucleus, inhibiting its upregulation of genes controlled by E-box enhancer elements (including those encoding PER/CRY and REV-ERBα). We write PER/CRY self-inhibition as:
(3)

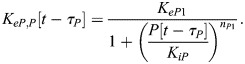


For simplicity, we model changes in REV-ERBα expression in the same manner as PER/CRY. Because both genes are regulated by E-box enhancers, we assume the expression terms are proportional to one another:
(4)

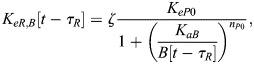

(5)

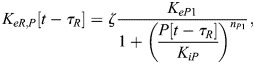
where *ζ* is a proportionality constant and *τ*_*R*_ is the delay between REV-ERBα transcription and changes in nuclear REV-ERBα.

Furthermore, we assume that nuclear YAP/TAZ, [*Y*_*nuc*_], and nuclear MRTF, [*M*_*nuc*_], independently regulate the expression of circadian proteins via TEADs and SRF, respectively. In reality, these processes might not be fully independent of the nuclear concentrations of BMAL1, PER/CRY and REV-ERBα, but sufficient mechanistic details are not available to justify a particular functional dependence. These YAP/TAZ and MRTF-dependent expression terms – *K*_*eB*2_, *K*_*eP*2_ and *K*_*eR*2_ – are written as sums of Hill equations:
(6)

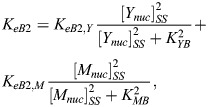

(7)

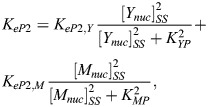

(8)

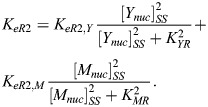


For each of these expression functions in Eqns 6, 7 and 8, the Hill coefficient was fixed at 2, as it was not found to affect the qualitative behavior of our model (Fig. S1). It should be noted that [*Y*_*nuc*_]_*SS*_ and [*M*_*nuc*_]_*SS*_ in Eqns 6, 7 and 8 are steady-state values; it is implicitly assumed that the mechanotransduction signaling pathways have reached steady state prior to the start of our simulations.

We write out the full set of DDEs, including decay terms for each species individually:
(9)

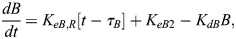

(10)

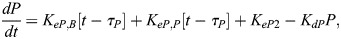

(11)

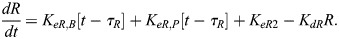


For a direct comparison of our model to experimental measurements of luminescence from cells expressing a PER2 promoter-driven luciferase reporter (PER2::Luc) (Yoo et al., 2004; Xiong et al., 2022), we explicitly modeled luciferase dynamics. Assuming that the expression of luciferase scales directly with PER2 expression and that cytosolic luciferase decays with rate *K*_*dL*_ (measured in Feeney et al., 2016), its dynamics are given by integrating the following expression:
(12)

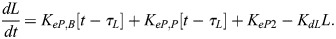


Altogether, this circadian clock model involves 28 free parameters as summarized in Table S1. Baseline values were chosen to match the single DDE system reported by Lema et al. (2000) or experimentally measured delays (Yagita et al., 2001, 2002; Lee et al., 2001; Tamaru et al., 2003), or they were empirically set from early testing of the model.

### Model calibration

**Fig. 2.**
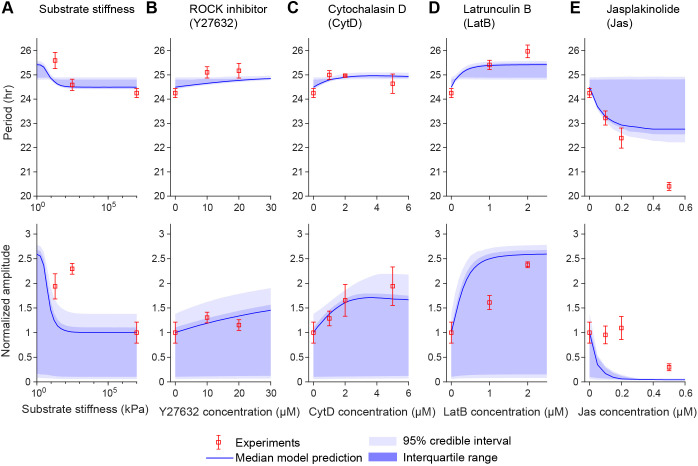
**Bayesian parameter estimation for the mechanotransduction–circadian model.** (A–E) Predicted oscillation period (top) and amplitude (bottom) in five experiments from Bayesian parameter estimation. Experimental data from Xiong et al. (2022) and model values are plotted for the effects of substrate stiffness (A), ROCK inhibitor (Y27632) treatment (B), cytochalasin D treatment (C), latrunculin B treatment (D) and jasplakinolide treatment (E). Experimental error bars denote s.d. of *n*=4 for each substrate stiffness, *n*=3 for all other treatment conditions. For the model, the darker blue region indicates interquartile range and the lighter blue region spans the 95% credible interval (region from the 2.5 percentile to 97.5 percentile), based on sampling from the posterior distributions for parameter values. To compute the estimates in this figure, 1000 samples were generated for each sequence of tests. In all cases, amplitude is normalized to that associated with the control condition (untreated cells on glass).

We assessed model sensitivity to each parameter by computing the total-order Sobol’ indices associated with the oscillation period and amplitude of the luciferase reporter (Fig. S2). Although the amplitude of the reporter was mostly sensitive to a few parameters (Fig. S2B), the oscillation period was sensitive to all free parameters in our model (Fig. S2A). Accordingly, we estimated all free parameters, including circadian parameters, MRTF transport parameters and inhibitor treatment-related parameters, rather than fix any at arbitrary default values. YAP/TAZ-related parameters were fixed to the values previously calibrated to experimental data (Scott et al., 2021). Given the inherent variability in biological systems, we estimated probability distributions rather than single values for all parameters in Tables S1 and S4 using Bayesian parameter estimation (Linden et al., 2022). We utilized measurements of PER2::Luc mouse primary fibroblasts reported by Xiong et al. (2022) to calibrate our model (Table S6). We minimized the error between model predictions and the experimental measurements for circadian oscillations after changing substrate stiffness (Fig. 2A) or treating cells with a ROCK inhibitor (Y27632) (Fig. 2B), cytochalasin D (Fig. 2C), latrunculin B (Fig. 2D) or jasplakinolide (Fig. 2E). These treatments introduce distinct effects on the cytoskeleton or associated signaling pathways while only introducing five additional free parameters to the model. Overall, simulations using parameters sampled from their posterior distributions (Fig. S2C) provided a reasonable fit to the experimental data (Fig. 2A–E). More details on sensitivity analysis and parameter estimation are given in the Materials and Methods (see section ‘Sensitivity analysis and parameter estimation').

### Mechanical factors alter the stability and dynamics of circadian oscillations

We examined the behavior of our model using the set of maximum *a posteriori* parameters from Bayesian parameter estimation (Tables S1 and S4). This corresponds to a single representative cell that exhibits behavior close to the mean population measurements from the work of Xiong et al. (2022).

**Fig. 3.**
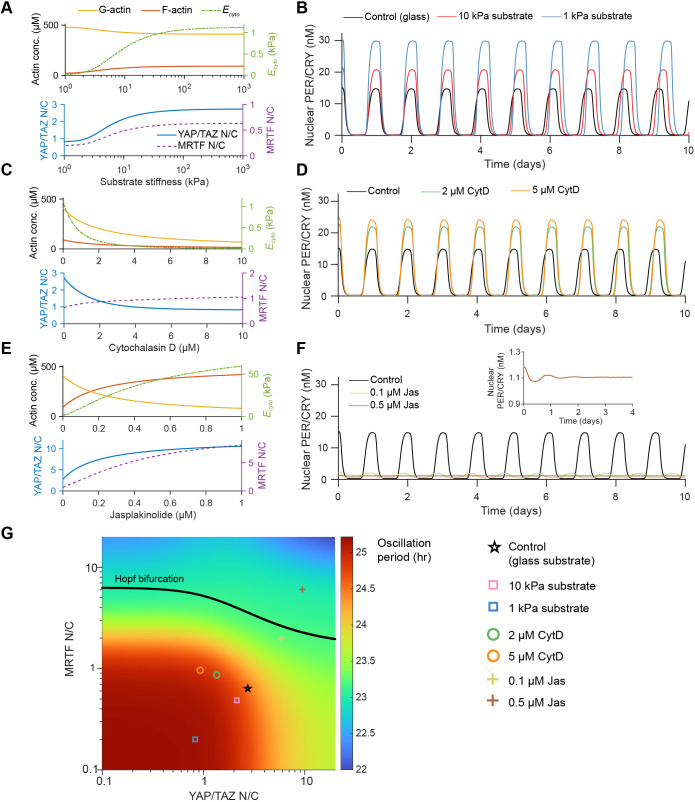
**Changes to circadian oscillations due to altered cytoskeletal activity.** (A) Substrate-stiffness-dependent changes in actin, cytosolic stiffness (*E*_*cyto*_), the YAP/TAZ nuclear-to-cytosolic (N/C) ratio and the MRTF N/C ratio. (B) Substrate-stiffness-dependent changes to oscillations in nuclear PER/CRY. (C) Cytochalasin D-induced changes to actin, cytosolic stiffness, and N/C ratios of YAP/TAZ and MRTF. Key as shown in A. (D) Cytochalasin D (CytD)-induced changes to oscillations in nuclear PER/CRY. (E) Jasplakinolide-induced changes to actin, cytosolic stiffness, and N/C ratios of YAP/TAZ and MRTF. F-actin increases dramatically upon jasplakinolide treatment, leading to substantial nuclear accumulation of YAP/TAZ and MRTF. Key as shown in A. (F) Jasplakinolide (Jas)-induced changes to oscillations in nuclear PER/CRY. Increased actin polymerization leads to a decrease in the oscillation period along with a decay in circadian oscillations (inset). (G) Bifurcation diagram showing the location of the Hopf bifurcation and the dependence of the circadian oscillation period on nuclear YAP/TAZ and MRTF. All points below the Hopf bifurcation curve correspond to sustained oscillations, whereas points above exhibit decaying oscillations. Individual markers plotted in the YAP/TAZ–MRTF phase plane correspond to the test cases shown in panels A–F. Conc., concentration.

We first tested the effects of changing substrate stiffness. We found that F-actin, cytosolic stiffness, nuclear YAP/TAZ and nuclear MRTF all increase consistently as a function of substrate stiffness (Fig. 3A). In turn, we observed marked changes to circadian oscillations (Fig. 3B); as substrate stiffness increases, the period and amplitude of circadian oscillations also increase, but the overall oscillatory nature remains consistent. These findings agree well with the data used for model calibration (Xiong et al., 2022) and validate the coupling between our mechanotransduction module and the circadian module.

Next, we investigated how two different cytoskeleton-targeting drugs, cytochalasin D and jasplakinolide, affect circadian oscillations. Cytochalasin D acts by capping existing actin filaments and inducing dimerization of G-actin. We modeled this in a similar manner to Wakatsuki et al. (2001), as fully described in the Materials and Methods and in Table S5. We observed that, because cytochalasin D treatment decreases the concentration of both F-actin and G-actin, it induces opposite effects on nuclear YAP/TAZ and MRTF (Fig. 3C). Specifically, nuclear YAP/TAZ decreases whereas nuclear MRTF increases due to the G-actin dimerization, which is in agreement with the results of previously published experiments (Finch-Edmondson and Sudol, 2016; Miralles et al., 2003). Jasplakinolide stabilizes actin filaments and also induces polymerization (Bubb et al., 2000). We modeled this by assuming that the polymerization rate increases and the depolymerization rate decreases as a function of jasplakinolide concentration (Eqns 28, 29; Table S5); as a result, F-actin accumulates in the cytosol, leading to increased levels of YAP/TAZ and MRTF in the nucleus (Fig. 3E).

As expected, we found that these inhibitor treatments have distinct effects on circadian oscillations. Similar to the effects of lower substrate stiffness, cytoskeletal inhibition by cytochalasin D leads to increased circadian oscillation period and amplitude (Fig. 3D). Conversely, when actin polymerization is enhanced via jasplakinolide treatment, YAP/TAZ and MRTF accumulate in the nucleus, leading to a slight decrease in oscillation period and rapidly decaying oscillations (Fig. 3F). This suggests that circadian oscillations are robust at lower levels of nuclear YAP/TAZ and MRTF, but upon accumulation of these factors in the nucleus, oscillations can decay over time. The location of this transition from stable amplitude to damped oscillations (the Hopf bifurcation) in the YAP/TAZ–MRTF plane is shown in Fig. 3G.

This general behavior, in which a Hopf bifurcation occurs for higher nuclear concentrations of YAP/TAZ and MRTF, is not exclusive to the single set of parameters used here. We found that this feature of the model is conserved for wide range of *K*_*dB*_, *K*_*dP*_ and *K*_*dR*_ (Fig. S3).

### Capturing of population-level variability in circadian oscillations

We next sought to capture population-level variability in circadian oscillations in our model. To incorporate population-level features, we focused on cell-to-cell variation of kinetic parameters. Taking inspiration from computational studies in cardiomyocytes (Ni et al., 2018; Kernik et al., 2019) and stochastic models of genetic oscillators (Veliz-Cuba et al., 2015), we represented this natural variability in kinetic parameters by generating a model cell population in which individual cells have kinetic parameters drawn from probability distributions. These distributions were directly adopted from the posterior distributions derived from Bayesian parameter estimation or, in the case of fixed parameters in the YAP/TAZ model, were assumed to be log normal (see Materials and Methods section ‘Simulation of cell populations’; Fig. S2C). Furthermore, to capture additional sources of measurement noise, we added Gaussian white noise to each time series after solving the DDEs.

**Fig. 4.**
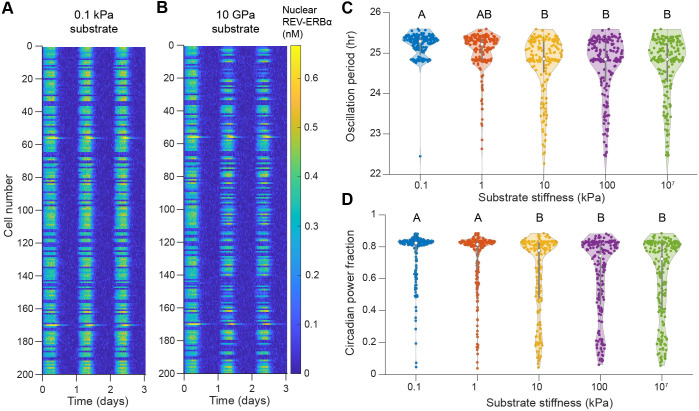
**Behavior of circadian oscillations in model cell populations with variability.** (A) Kymograph depicting oscillations in nuclear REV-ERBα for 200 model cells on a soft (0.1 kPa) substrate. Cells exhibit consistent circadian oscillations, and variability is observed at the level of the population. (B) Kymograph depicting oscillations in nuclear REV-ERBα for 200 model cells on glass (10 GPa). Most cells show consistent, lower magnitude oscillations; in some cases, oscillations are weaker or nonexistent. (C,D) Distribution of oscillation period (C) and circadian power fraction (D) for cell populations (200 model cells each) on different substrate stiffnesses, as indicated. Violin plots show the distribution of data, with the central points and error bars marking the median and interquartile range, respectively. Compact letter display is used to denote statistical significance, where groups sharing a letter are statistically similar according to one-way ANOVA followed by Tukey's post hoc test with a significance threshold of *P*=0.05. Violin plots were generated using Violinplot in MATLAB (https://github.com/bastibe/Violinplot-Matlab).

We first verified that simulations using this modified model still captured the overall trends seen at the single-cell level (Fig. 3) while displaying increased variability. In this case, we analyzed oscillations in REV-ERBα rather than PER/CRY for a direct comparison with the population-level data from the work of Abenza et al. (2023). Plotting population REV-ERBα dynamics as a kymograph, we observed that circadian oscillations appear to be stronger in cells on a soft substrate compared to those in cells on glass (Fig. 4A,B), similar to the decreased oscillation amplitude observed on stiff substrates in the single-cell case (Fig. 3B). In further agreement with our single-cell observations, we found that cells on stiffer substrates exhibit decreased oscillation period on average (Fig. 4C).

To quantify disruptions to regular circadian oscillations, we used a metric called the circadian power fraction (originally defined in Abenza et al., 2023), which ranges from 0 (no circadian oscillations) to 1 (perfect sinusoidal circadian oscillations). This metric corresponds to the fraction of the circadian power spectrum contained within a close window of the population-level average frequency (as detailed in the Materials and Methods section ‘Calculation of the circadian power fraction’). We observed that lower substrate stiffnesses are generally associated with higher circadian power fractions, whereas increased accumulation of YAP/TAZ and MRTF in the nucleus on stiff substrates results in lower circadian power fractions (Fig. 4D). These lower circadian power fractions indicate significantly disrupted oscillations, which manifest as weaker oscillations that are drowned out by Gaussian noise in our simulations (Fig. 4B). We note that Abenza et al. did not observe the large increase in circadian power fraction on softer substrates that we report here (Abenza et al., 2023); however, the average YAP/TAZ nuclear-to-cytosolic (N/C) ratio also remains elevated for cells on soft substrates in their study, perhaps indicating that their cells remained in a mechanically active state not accounted for in our model. Alternatively, other factors could contribute to changes in the expression of circadian proteins in this case; this option is further explored below.

### Nuclear abundances of YAP/TAZ and MRTF correlate with the strength of circadian oscillations across treatment conditions

Next, we considered how other changes in the mechanical environment or distinct pharmacological treatments that disrupt the cytoskeleton can impact the circadian power fraction. We tested the set of conditions examined experimentally by Abenza et al. (2023), including changes in substrate stiffness, cell density or cell adhesion area, as well as treatment with cytochalasin D, latrunculin A or blebbistatin. Although several of these treatments were considered in our model calibration and initial testing, here we directly leveraged the model against this separate set of experiments, and we mainly focused on the strength of oscillations at the cell population level. The assumed effects of each treatment are given in Table S5, with further details in the Materials and Methods. In brief, increased cell density and restriction of cell adhesion area (1600 μm^2^ or 900 μm^2^ micropatterns) were both assumed to reduce the amount of FAK phosphorylation due to less cell–substrate contact (Eqns 31, 32). Increased cell density was also assumed to increase the YAP/TAZ phosphorylation rate due to activation of LATS (Table S5). Latrunculin A and blebbistatin treatment decreased the F-actin polymerization rate and the formation of active stress fibers, respectively (Table S5).

**Fig. 5.**
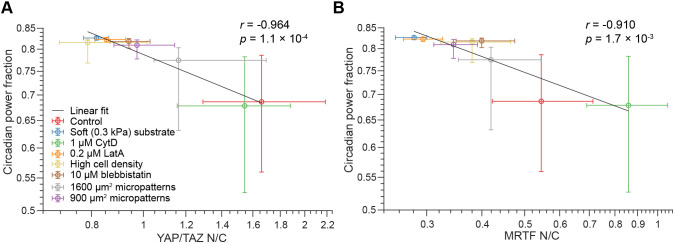
**Correlation between circadian power fraction and the N/C ratios of YAP/TAZ or MRTF.** (A,B) Populations of model cells were subjected to the treatments tested by Abenza et al. (2023), and circadian power fraction was plotted against YAP/TAZ N/C ratio (A) and MRTF N/C ratio (B). Data points denote median values and error bars denote the range from the 40th to the 60th percentile for 200 model cells. In each case, Pearson's correlation coefficient (*r*) was assessed for the correlation between median power fraction and median N/C ratio on the log scale. Values of *r* and the *P*-value associated with the null hypothesis, *r*=0, are included on each graph. To generate distinguishable colors for each condition, we used the linspecer tool in MATLAB (https://www.mathworks.com/matlabcentral/fileexchange/42673-beautiful-and-distinguishable-line-colors-colormap). CytD, cytochalasin D; LatA, latrunculin A.

As expected, we found that all of these treatments decrease nuclear YAP/TAZ and MRTF on average, with the exception of cytochalasin D treatment, which increases the nuclear concentration of MRTF, as described above (Fig. 5). Given the results described above, we would expect any treatments decreasing nuclear YAP/TAZ and/or MRTF to enhance circadian oscillations. Indeed, in all cases except cytochalasin D treatment, we observed an increase in the median circadian power fraction (Fig. 5).

In Abenza et al. (2023), the authors report a significant correlation between the YAP/TAZ N/C ratio and the circadian power fraction, but no such correlation for the MRTF N/C ratio. In agreement with their findings, we observed a strong correlation between the circadian power fraction and nuclear YAP/TAZ (Fig. 5A). However, we also found a significant correlation of the circadian power fraction with nuclear MRTF (Fig. 5B). Notably, this correlation is weaker than that with YAP/TAZ (see correlation coefficient statistics in Fig. 5). Additionally, we note that Abenza et al. (2023) report that cells exhibit low circadian power fractions even on soft substrates. Accordingly, we postulated that an additional mechanism might disrupt circadian oscillations on soft substrates. We tested this by increasing the baseline expression of BMAL1 and PER/CRY in cells on a soft substrate and observed that the correlation with MRTF nuclear abundance was no longer significant, whereas the correlation with nuclear YAP/TAZ remained significant (Fig. S4). This higher sensitivity of circadian oscillation amplitude to changes in YAP/TAZ is consistent with the fact that, among mechanotransduction–circadian coupling parameters, circadian oscillation amplitude is most sensitive to *K*_*eP2,Y*_ (Fig. S2B). Furthermore, we could clearly see that oscillation amplitude changes more quickly with the YAP/TAZ N/C ratio than with the MRTF N/C ratio over the YAP/TAZ–MRTF phase plane for the region corresponding to the conditions tested here (Fig. S3B).

### Computational predictions establish that mutations in YAP or *LMNA* can significantly disrupt circadian oscillations

**Fig. 6.**
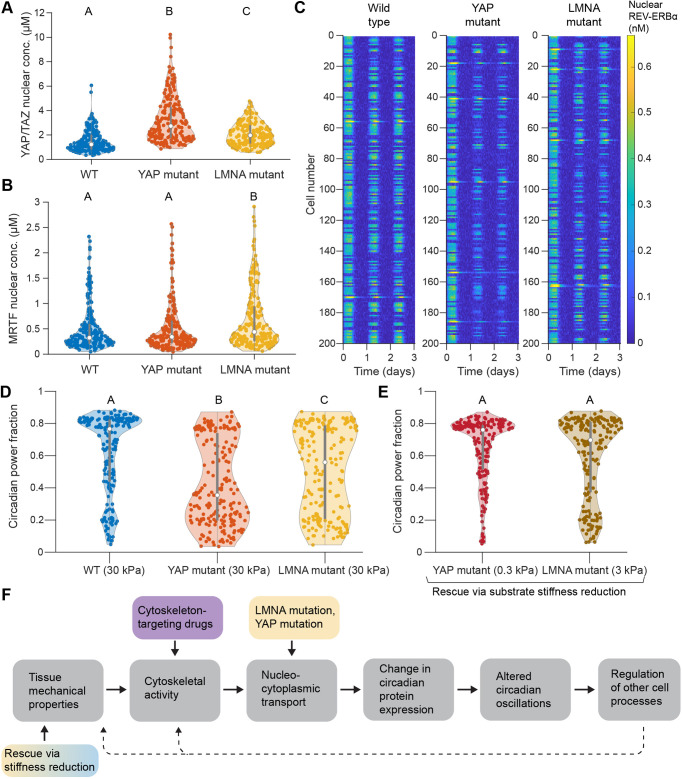
**Effects of YAP or lamin A mutations on circadian oscillations.** (A,B) Nuclear concentrations (conc.) of YAP/TAZ (A) and MRTF (B) compared across a wild-type (WT) population, YAP mutant population and *LMNA* mutant population, all on 30 kPa substrates. (C) Kymographs depicting the dynamics of nuclear REV-ERBα for a wild-type population, YAP mutant population and *LMNA* mutant population, all on 30 kPa substrates. Each plot shows 200 cells over 3 days. (D) Circadian power fraction compared across wild-type, YAP mutant and *LMNA* mutant cells on 30 kPa substrates. (E) Rescue of normal circadian oscillations (circadian power fraction) in YAP mutant and *LMNA* mutant cells via reduction of substrate stiffness, as indicated. Violin plots in A, B, D and E show the distribution of data, with the central points and error bars marking the median and interquartile range, respectively, for populations of 200 cells each. Compact letter display is used to denote statistical significance, where groups sharing a letter are statistically similar according to one-way ANOVA followed by Tukey's post hoc test with a significance threshold of *P*=0.05. Comparisons in D and E were conducted across all five groups. Violin plots were generated using Violinplot in MATLAB (https://github.com/bastibe/Violinplot-Matlab). (F) Summary of the causal flow from cell mechanotransduction to changes in the circadian clock. Upper colored boxes denote disruptions to mechanotransduction explored at different points in this article, either due to cytoskeleton-targeting drugs or mutations in YAP or *LMNA*. The lower box depicts the rescue of normal circadian oscillations in mutant cells via a reduction in substrate stiffness. Dashed arrows indicate possible feedback from the circadian clock to cell and tissue mechanics.

Both our single-cell model and our population-level model predict that YAP/TAZ and MRTF nuclear concentrations modulate the strength and stability of circadian oscillations (Figs 3G, 4 and 5). We therefore reasoned that mutations affecting the associated pathways for nucleo-cytoplasmic transport could strongly impact circadian oscillations. We first tested this by considering overexpression of the YAP mutant 5SA-YAP; such overexpression in fibroblasts has previously been found to result in a large reduction in the circadian power fraction (Abenza et al., 2023). The 5SA-YAP mutant has several phosphorylation sites removed, causing it to accumulate at abnormal levels in the nucleus. We tested this condition in our model by introducing a new species, 5SA-YAP, which behaved the same as wild-type YAP/TAZ but had a phosphorylation rate equal to zero (Table S5). In these tests, the amount of wild-type YAP/TAZ and mutant YAP were each equal to the amount of YAP/TAZ in control cells, resulting in twice the overall YAP/TAZ per cell. Due to the combined effects of overexpression and decreased phosphorylation, YAP/TAZ nuclear concentration significantly increased compared to that in wild-type cells (Fig. 6A).

Separately, we tested possible effects of mutations in *LMNA*, the gene controlling expression of lamin A. We considered a combination of two alterations to the model due to this mutation, aiming to mimic states relevant to laminopathies. Given that laminopathy-associated lamin mutants show distinct differences in their phosphorylation states (Torvaldson et al., 2015; Cenni, 2005; Mitsuhashi et al., 2010; Swift et al., 2013), we first assumed that the mutated lamin A was constitutively active (dephosphorylated). We additionally assumed that mutations in lamin A could result in increased nuclear permeability due to changes in localization of NPCs or in overall nuclear shape (Dutta et al., 2018). We set the lamin A phosphorylation rate to zero and doubled the opening rate of NPCs (Table S5), leading to increased levels of both YAP/TAZ and MRTF in the nucleus (Fig. 6A,B), in agreement with recent experimental data (Owens et al., 2020). Although the effects of such a mutation were informed by the above studies, this condition is not meant to directly represent any one given lamin A mutation, but rather to predict the effects of some possible lamin-related defects. We ensured the robustness of our results by testing different values of mutation-related parameters for *LMNA* and 5SA-YAP, as shown in Fig. S5.

We found that on 30 kPa substrates, both mutants show altered BMAL1 dynamics, with significantly weaker oscillations compared to those in wild-type cells (Fig. 6C). The circadian power fraction decreases distinctly in each case, with the effect being relatively stronger for 5SA-YAP cells (Fig. 6D). This is expected, given the dramatic increase in nuclear YAP/TAZ due to overexpression of 5SA-YAP, compared to the milder increases in both YAP/TAZ and MRTF nuclear concentrations observed for the lamin A mutant (Fig. 6A,B). This strong influence of 5SA-YAP overexpression on circadian power fraction agrees qualitatively with the findings of Abenza et al. (2023); for ease of comparison, we directly compare our kymographs and circadian power fractions with those from experiments in Fig. S6.

Our previous population-level tests establish that lower amounts of mechanical activation (e.g. lower substrate stiffness, treatment with cytoskeletal inhibitors) generally lead to increases in the circadian power fraction (Figs 4 and 5). Accordingly, we tested whether a reduction in substrate stiffness might rescue circadian oscillations for either mutant. Remarkably, we found that decreasing substrate stiffness to 3 kPa for the lamin A mutant, or to 0.3 kPa for the 5SA-YAP mutant, resulted in a power fraction statistically similar to that of wild-type cells on a 30 kPa substrate (Fig. 6E). This provides a prediction that can readily be tested experimentally and indicates a possible compensatory mechanism to counteract perturbations to circadian oscillations in laminopathies.

## DISCUSSION

Circadian rhythms are exhibited by most organisms on earth and appear across biological scales, from oscillations in gene expression in single cells to changes in organism-wide features such as body temperature. These changes in turn regulate physiological processes such as sleep–wake cycles and metabolic activity in healthy organisms. However, disruptions of regular circadian oscillations are associated with various diseases ranging from cancer (Shafi and Knudsen, 2019) to diabetes (Mason et al., 2020), placing a high priority on research efforts to understand the regulation of circadian rhythms from the cell to the organism level.

Several recent studies have found that local environmental cues such as substrate stiffness and cytoskeletal activity can modulate the cell circadian clock (Abenza et al., 2023; Xiong et al., 2022; Williams et al., 2018; Yang et al., 2017). However, previous models of circadian oscillations in single cells do not include any effects on the expression of circadian proteins due to such factors. In our minimal model, we propose that nuclear YAP/TAZ and MRTF can alter the expression of BMAL1, PER/CRY and REV-ERBα independently of other components of the circadian clock. As supported by recent experimental work (Abenza et al., 2023; Xiong et al., 2022), this change in expression seems to be mediated by a combination of TEADs (in the case of YAP/TAZ) and SRF (in the case of MRTF). Although many of the mechanistic details behind this mechanotransduction–circadian coupling remain unclear, our calibrated model fits well to the experimental data and agrees with experimental measurements of cell populations. This suggests that YAP/TAZ and MRTF integrate different mechanical effects on cells – including actin polymerization, cytosolic stiffness and myosin activity – to provide specific regulatory cues to the circadian clock. These mechanosensitive effects could well extend to other circadian proteins such as RORc and likely differentially regulate different isoforms of PER and CRY. Such details are neglected in the minimal modeling approach we adopt here but could be well-addressed by integrating future experimental results with more detailed models.

From the main experimental work we draw upon here (Abenza et al., 2023; Xiong et al., 2022), the individual roles of YAP/TAZ and MRTF-mediated mechanotransduction remain unclear. Whereas the work of Xiong et al. (2022) focuses exclusively on MRTF-mediated activation of SRF and how this leads to changes in the expression of circadian proteins, results reported by Abenza et al. (2023) suggest that YAP/TAZ mediates changes in both BMAL1 and PER/CRY expression. In fact, Abenza et al. (2023) show that YAP/TAZ nuclear levels correlate with the circadian power fraction but MRTF nuclear levels do not. Results from our simulations indicate that these findings need not be contradictory. Although we assume that both YAP/TAZ and MRTF both mediate disruptions to the circadian clock, we find that YAP/TAZ correlates more strongly with the circadian power fraction (Fig. 5). However, unlike Abenza et al. (2023), we do observe a significant correlation between the MRTF N/C ratio and circadian power fraction. This difference appears to be related to the assumed effect of substrate stiffness on the circadian power fraction; our model predicts a larger increase in power fraction for cells on soft substrates than that observed by Abenza et al. (2023). Indeed, if we assume that a separate mechanism leads to increased expression of BMAL1 and PER/CRY in cells on soft substrates, the correlation with MRTF nuclear abundance is no longer significant, whereas the correlation with nuclear YAP/TAZ remains significant (Fig. S4).

The overall trend of weaker circadian oscillations for increased mechanical activation agrees well with previously reported measurements made in epithelial cells; however, the same study found the opposite trend in mouse fibroblasts (Williams et al., 2018). This apparently disagrees with the trend observed in mouse fibroblasts by Xiong et al. (2022). However, we note that the study by Williams et al. only assessed cell responses to softer hydrogels in three-dimensional (3D) culture (Williams et al., 2018), whereas Xiong et al. made all measurements on two-dimensional (2D) substrates (Xiong et al., 2022). Aside from tissue- or organism-specific differences in fibroblast behavior, the difference in observations could be attributed to the well-established differences between cell behavior in 2D versus 3D culture (Baker and Chen, 2012; Duval et al., 2017). This could be investigated in future modeling work examining the effects of different spatial stimuli on cell mechanotransduction (Scott et al., 2021).

Our model predicts that certain mutations in YAP/TAZ or lamin A could significantly disrupt circadian oscillations in cell populations (Fig. 6). In these cases, increased nuclear accumulation of YAP/TAZ and/or MRTF leads to perturbations to the circadian network such that oscillations are much weaker. This agrees well with experimental measurements in which overexpression or knockout of lamin A leads to significant changes in the expression of some circadian proteins (Roskell, 2019). Furthermore, these findings have natural implications for laminopathies – diseases that are characterized by mutations in *LMNA*. We specifically considered effects of *LMNA* mutations matching those previously found for laminopathy-associated mutations, including defects in lamin phosphorylation (Torvaldson et al., 2015) and changes in NPC localization and nuclear shape (Dutta et al., 2018). Therefore, disruptions to the cell circadian clock could be one underappreciated component of disease state in laminopathies, as also posited in other recent work (Briand and Collas, 2018; Roskell, 2019). For instance, due to the well-known role of the circadian clock in regulating metabolic processes (Sahar and Sassone-Corsi, 2012), any such disruptions to the circadian clock could contribute to altered metabolism commonly observed in laminopathy patients (Decaudain et al., 2007; Guénantin et al., 2014).

Our model might provide insights into other disease states as well. For instance, tissue stiffening is a common hallmark of diseases from cancer to atherosclerosis (Chin et al., 2016; Palombo and Kozakova, 2016). As predicted by our model (Fig. 4), increased substrate stiffness could locally perturb circadian oscillations in cells. Given the host of cellular processes regulated by circadian cues, any such disruptions could have large effects on overall cell function. Additionally, the results of our *in silico* rescue experiment (Fig. 6E) suggest that changes in the local mechanical properties of tissue could serve to counteract disruptions to normal circadian oscillations in diseased cells. For instance, a 10-fold reduction in stiffness (from 30 kPa to 3 kPa) is sufficient to rescue normal circadian oscillations in our simulated *LMNA* mutant. This may provide an inherent biological strategy to recover normal oscillations in diseases such as laminopathies. Overall, these findings suggest that mutations affecting nuclear transport render cells especially vulnerable to dysfunctions in circadian oscillations upon tissue stiffening.

Other factors currently not considered in our model could have important effects on the nuclear translocation of YAP/TAZ and MRTF, thereby influencing any downstream disruptions to circadian oscillations. For instance, the disruptive effect of substrate stiffness on circadian oscillations could be slightly alleviated for cells in 3D culture conditions, as previous modeling work shows that cells simulated in 2D culture can exhibit higher YAP/TAZ N/C ratio compared to cells in 3D culture (Scott et al., 2021). Moreover, changes in cell shape are known to influence YAP/TAZ nuclear translocation (Scott et al., 2021; Eroumé et al., 2021). Finally, many other pathways such as Ca^2+^ signaling cascades are known to play important roles in determining the YAP/TAZ N/C ratio (Khalilimeybodi et al., 2023). These various factors provide natural areas for future extensions of our model.

As it stands, the coupling in our model goes only one way; that is, YAP/TAZ and MRTF alter the expression of circadian proteins. In reality, changes to the circadian oscillation period can induce other changes in cell physiology that could couple back to the mechanical state of cells and/or tissue, creating a closed feedback loop (Fig. 6F). For instance, the circadian clock plays an important regulatory role in extracellular matrix remodeling, controlling processes ranging from collagen deposition to matrix metalloproteinase activity (Dudek et al., 2023; Hahn and Sundar, 2023). Rather than assuming that the cell reaches mechanical steady state prior to the start of the simulation, YAP/TAZ and MRTF dynamics could readily be integrated within our equations. In the future, this approach would give us a unique opportunity to probe the complex interplay between circadian clocks, mechanotransduction and disease states.

## MATERIALS AND METHODS

### Numerical implementation

Our code is implemented in MATLAB 2023 and is freely available on GitHub (https://github.com/RangamaniLabUCSD/MechanoCircadian; Francis, 2024b). Given the set of treatment conditions (substrate stiffness, cell density, inhibitor concentrations), we algebraically solved for the steady-state concentrations of nuclear YAP/TAZ and nuclear MRTF. These values were then used to compute *K*_*eB*2_, *K*_*eP*2_ and *K*_*eR*2_ (Eqns 6, 7 and 8), and the resulting system of DDEs (Eqns 9, 10 and 11) was solved using dde23 in MATLAB. As initial conditions in dde23, we used *B*_0_=5B*, *P*_0_=0.2P* and *R*_0_=R*, where B*, P* and R* were respectively the steady-state BMAL1, PER/CRY and REV-ERBα values associated with a given treatment condition. The luciferase concentration, *L*(*t*), was then computed by integrating Eqn 12 using ode15s in MATLAB, with the initial condition *L*_0_=0.

### Sensitivity analysis and parameter estimation

We conducted a global parametric sensitivity analysis using the UQLab framework in MATLAB (Marelli and Sudret, 2014). Treating the luciferase oscillation period and amplitude as quantities of interest, we estimated the total-order Sobol’ indices associated with parameters from Tables S1 and S4. These indices represent the total effect of a parameter on the given output, including first-order effects and all higher-order effects arising from interactions with other parameters. We only considered the case of untreated cells on glass (10 GPa stiffness) and did not test the sensitivity to any inhibitor-related parameters. Sobol’ indices were computed using the default Monte Carlo estimators in UQLab with a sample size of 50,000. Marginal distributions were uniform for all parameters over the fitting ranges specified in Tables S1 and S4.

We completed our model calibration using Bayesian parameter estimation in UQLab, yielding likelihood distributions for each parameter given the mean experimental measurements and associated errors. After computing the luciferase reporter dynamics as described above, we calculate a likelihood function by computing the product of error distributions at discrete time steps for each test. With 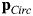 as a vector of free parameters, *k* as the number of experimental conditions and *N*_*i*_ as the number of time points evaluated for test *i*, the log-likelihood function 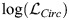 is:
(13)

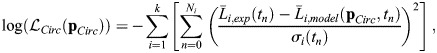
where 
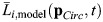
 and 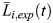 are the normalized luciferase dynamics in the model and experiments. For comparison to the experimental data, we time-shifted *L* to start at the second peak in the simulated dynamics (*t*_*peak*_), normalized to the amplitude of oscillations in the control case (*A*_*model*,*control*_), and subtracted the mean value of *L* for the current condition:
(14)

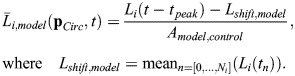


The experimental dynamics were reconstructed from the reported measurements of the period and amplitude of luminescence oscillations given by Xiong et al. (2022). For each condition, Xiong et al. measured the population average and standard deviation for the period and amplitude of oscillation, which we measured from their bar graphs using manual image analysis in ImageJ (https://imagej.net/software/imagej/) (Table S6). Treating the period (*T*_*i*,*exp*_) and amplitude (*A*_*i*,*exp*_) as normally distributed random variables, the relative luminescence over time is given by:
(15)

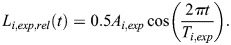


We generated instances of 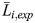 by sampling from the distributions for *T*_*i*,*exp*_ and *A*_*i*,*exp*_ for each condition. We assumed that the period and amplitude are normally distributed with mean and standard deviations from Table S6 and generated one million pairs of samples per condition. The mean 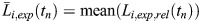 and standard devation *σ*_*i*_(*t*_*n*_)=std(*L*_*i*,*exp*,*rel*_(*t*_*n*_)) for the luminescence were then computed at discrete time points (every 1 h).

To better match the experimentally measured oscillation periods, we found it necessary to augment the above log-likelihood function by adding an extra penalty to any model deviations from the experimentally measured oscillation periods:
(16)

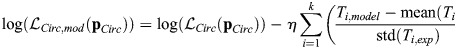
where *η* is a weighting factor determining the magnitude of the penalty term. We tested different values of this factor, finding good performance for *η*=100.

We sampled from the posterior distribution using Markov chain Monte Carlo (MCMC) in UQLab. Specifically, we used the built-in affine invariant ensemble algorithm with 60 walkers and 1000 total steps. Prior distributions were chosen to be uniform over the ranges specified in Tables S1 and S4. We assessed convergence of MCMC using the integrated autocorrelation time (IACT), which corresponds to the number of steps in the MCMC algorithm required for all walkers to decorrelate from their initial trajectory. We computed this using previously developed code (Wolff, 2004; Linden et al., 2022), finding an approximate value of 88 steps. As a conservative measure, we discarded the first 500 steps as burn-in when sampling from the posterior distributions. The posterior distributions for each parameter are plotted in Fig. S2C.

### Linear stability analysis

To assess which parameters naturally give rise to sustained circadian oscillations, we conducted a linear stability analysis. Numerical bifurcation analysis was conducted using DDE-BIFTOOL in MATLAB (Engelborghs et al., 2002). For this analysis, we used the maximum *a posteriori* (MAP) values as point estimates for each circadian parameter (Table S1) and treated the N/C ratios of YAP/TAZ and MRTF as bifurcation parameters. In this context, the Hopf bifurcation is defined by the transition of the rightmost (most positive/least negative) pair of eigenvalues from negative real values to positive real values (Fig. S3A). This corresponds to a supercritical Hopf bifurcation, in which damped oscillations transition to a stable limit cycle.

Given the known importance of degradation rates on circadian oscillations, we also examined the effects of *K*_*dB*_, *K*_*dP*_ and *K*_*dR*_ on the location of Hopf bifurcations and the period of oscillations (Fig. S3C–E).

### Equations for inhibitor treatments

Throughout this work, we considered several different treatments known to modify the cytoskeleton via distinct mechanisms. Cytochalasin D, latrunculin A and latrunculin B all have an overall inhibitory effect on actin polymerization, but they trigger distinct effects on MRTF versus YAP/TAZ nuclear transport (Finch-Edmondson and Sudol, 2016; Miralles et al., 2003), prompting careful consideration in our model.

Under standard conditions, actin polymerization can be written in the following form (see Tables S2 and S3 for full details):
(17)

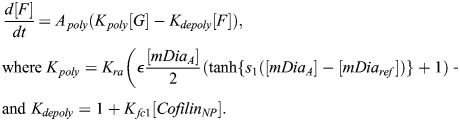


[*F*] is the concentration of F-actin and [*G*] is the concentration of G-actin. *A*_*poly*_ is a constant that sets the overall timescale, which can be arbitrarily set for the sake of the steady-state analysis here. Note that the value of [*F*] corresponds to the concentration of actin monomer units per volume; to convert this to the molecular concentration of filaments we would divide by the average length of actin filaments in the cell. Taking into account mass conservation ([*F*]+[*G*]=[*Actin*_*tot*_]), the steady-state value of [*F*] is:
(18)

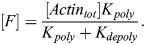


To model the action of cytochalasin D, latrunculin A and latrunculin B, we used a simplified version of the equations from Wakatsuki et al. (2001). In all cases, we assumed the inhibitor to be present in excess.

#### Cytochalasin D treatment

Cytochalasin D acts by capping existing actin filaments and inducing dimerization of G-actin. Accordingly, we considered additional differential equations for cytochalasin D-induced dimerization, [*CG*_2_], and cytochalasin D capping of F-actin, [*FC*]:
(19)

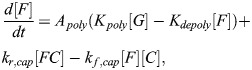

(20)

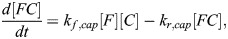

(21)

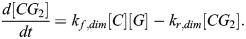


Note that, for simplicity, we assumed that the rate of dimerization scales with [*C*][*G*] rather than [*C*][*G*]^2^. This is equivalent to assuming that all cases of cytochalasin D/G-actin binding result in G-actin dimerization after the first binding event, capturing the same qualitative effect while yielding a simpler closed form expression for [*F*]. The corresponding steady-state concentration of F-actin can be readily solved for, assuming mass conservation ([*F*]+[*G*]+2[*CG*_2_]+[*FC*]=[*Actin*_*tot*_]):
(22)

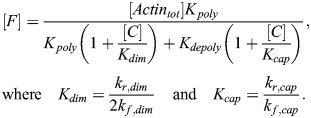
We assumed that dimerized G-actin in the case of cytochalasin D treatment no longer sequesters MRTF. Therefore, the relevant G-actin concentration [G] is given by:
(23)

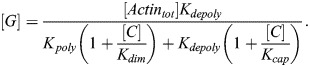


#### Latrunculin A and latrunculin B treatments

Latrunculin A and latrunculin B act by sequestering G-actin. Writing latrunculin concentration as [*L*] and the G-actin–latrunculin complex concentration as [*GL*], we have the following set of ODEs:
(24)

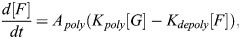

(25)

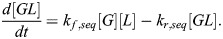


Accounting for conservation of mass ([*G*]+[*GL*]+[*F*]=[*Actin*_*tot*_]), the steady-state solution for F-actin and total G-actin can be written as:
(26)

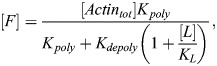

(27)

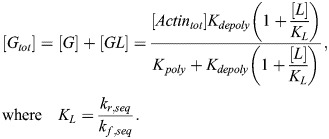


Note that the total G-actin, [*G*_*tot*_], is assumed to be the relevant concentration for MRTF sequestration and is therefore the quantity used to compute the amount of free MRTF in this case (see Table S2).

#### Jasplakinolide treatment

In the case of jasplakinolide treatment, in line with *in vitro* data (Bubb et al., 2000), we made the empirical assumption that actin polymerization rate increases as a function of jasplakinolide concentration [*J*]:
(28)

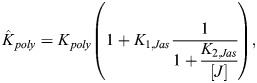

(29)

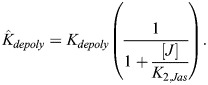


Note that in the general case, 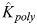 and 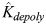 could have different sensitivities to jasplakinolide (*K*_2,*Jas*_ here), but existing data support these sensitivities being similar (Bubb et al., 2000). In this case, the new steady-state F-actin concentration is:
(30)

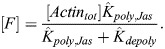


#### Other inhibitor treatments and contact area considerations

For all other inhibitor treatments, we assumed general Hill equations for concentration-dependent inhibition. The effects of all inhibitor treatments and mutations are summarized in Table S5.

In all cases from Abenza et al. (2023), we additionally considered the effects of changes in cell–substrate contact area, and we approximated contact area by tracing out the fluorescence microscopy images in figure 3D and figure S3A of their paper (Abenza et al., 2023). Control cells had contact area 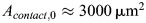, and any changes in contact area were captured by modulating the amount of FAK phosphorylation:
(31)

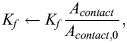

(32)

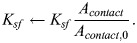


### Simulation of cell populations

Model populations of cells were generated by sampling parameter values from probability distributions. By default, all parameters in our YAP/TAZ mechanotransduction model were sampled from log-normal distributions. We selected parameter values from each distribution by first generating a normally distributed random number *r* using randn() in MATLAB, and then computing:
(33)

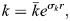
where *σ*_*k*_ is the standard deviation associated with the log-normal distribution of values for *k* and 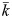 is the baseline value of the parameter. A value of *σ*_*k*_=0.2 was used for all parameters except for exponents, which were kept constant.

For all MRTF and circadian parameters fit using the Bayesian approach described above, their probability distributions were described by their posterior distributions (Fig. S2C). We randomly drew from these distributions by sampling from the MCMC chains after discarding the first 500 iterations as burn-in.

### Calculation of the circadian power fraction

We used the circadian power fraction as a metric for the quality of circadian oscillations, as defined by Abenza et al. (2023). In their paper, they measured circadian oscillations using the fluorescent sensor REV-VNP (Nagoshi et al., 2004) as an indicator for REV-ERBα expression, and so we computed the power fraction from our predictions of REV-ERBα dynamics. Before computing the power fraction, we added white Gaussian noise to individual REV-ERBα trajectories, assuming a signal-to-noise ratio of 5. We sampled the resulting signal every 15 min (sampling frequency *f*_*S*_=96 day^−1^) and then filtered it using a low-pass filter with the same settings as reported by Abenza et al. (2023) [filtfilt() in MATLAB with a=1 and b=(0.2, 0.2, 0.2, 0.2)] and scaled the dynamics by subtracting the mean and dividing by the standard deviation. We then computed the power spectrum, 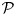, for each cell in the model population for a given condition. The power fraction for an individual cell was then given by:
(34)

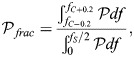
where *f*_*C*_ is the frequency associated with the maximum population-wide average power in the interval 0.7 day^−1^ to 1.3 day^−1^.

## Supplementary Material



10.1242/joces.261782_sup1 Supplementary information
