# Supplementary material for: Computational modeling establishes mechanotransduction as a potent modulator of the mammalian circadian clock
Source: J Cell Sci. 2024 Sep 9;137(17):jcs261782. doi: 10.1242/jcs.261782 (PMC11423814; doi:10.1242/jcs.261782)
Supplement: Supplementary information [file joces-137-261782-s1.pdf]

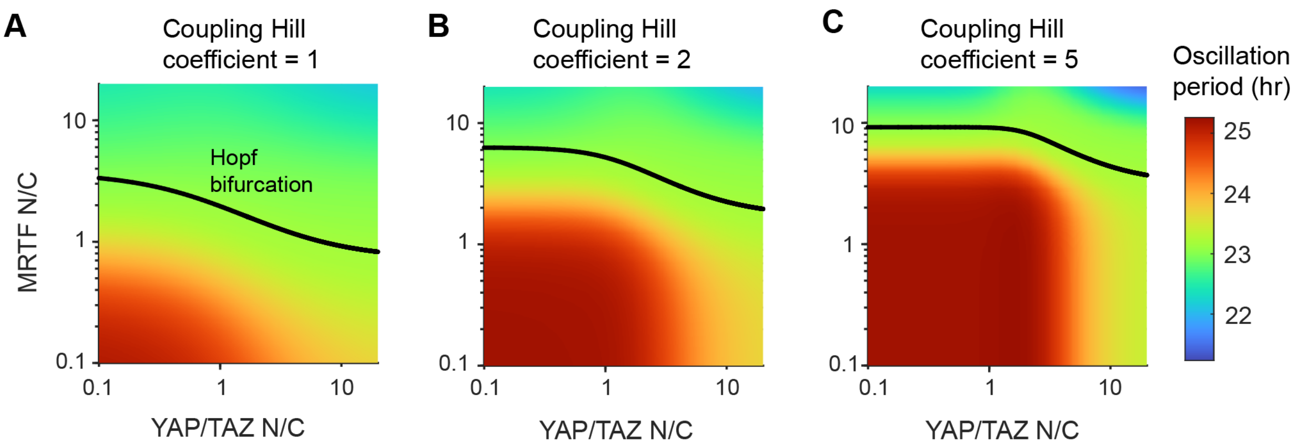

Fig. S1. Effect of Hill coefficient on mechanotransduction-circadian coupling. Oscillation period and Hopf bifurcation plotted over the MRTF-YAP/TAZ phase plane for different choices of Hill coefficient in Equations 6-8. For simplicity, the same Hill coefficient was used in all equations for each case, either 1 (A), 2 (B, same as all other tests in the paper), or 5 (C). Changing the Hill coefficient simply shifts the Hopf bifurcation line and oscillation period without changing the qualitative behavior of the system.

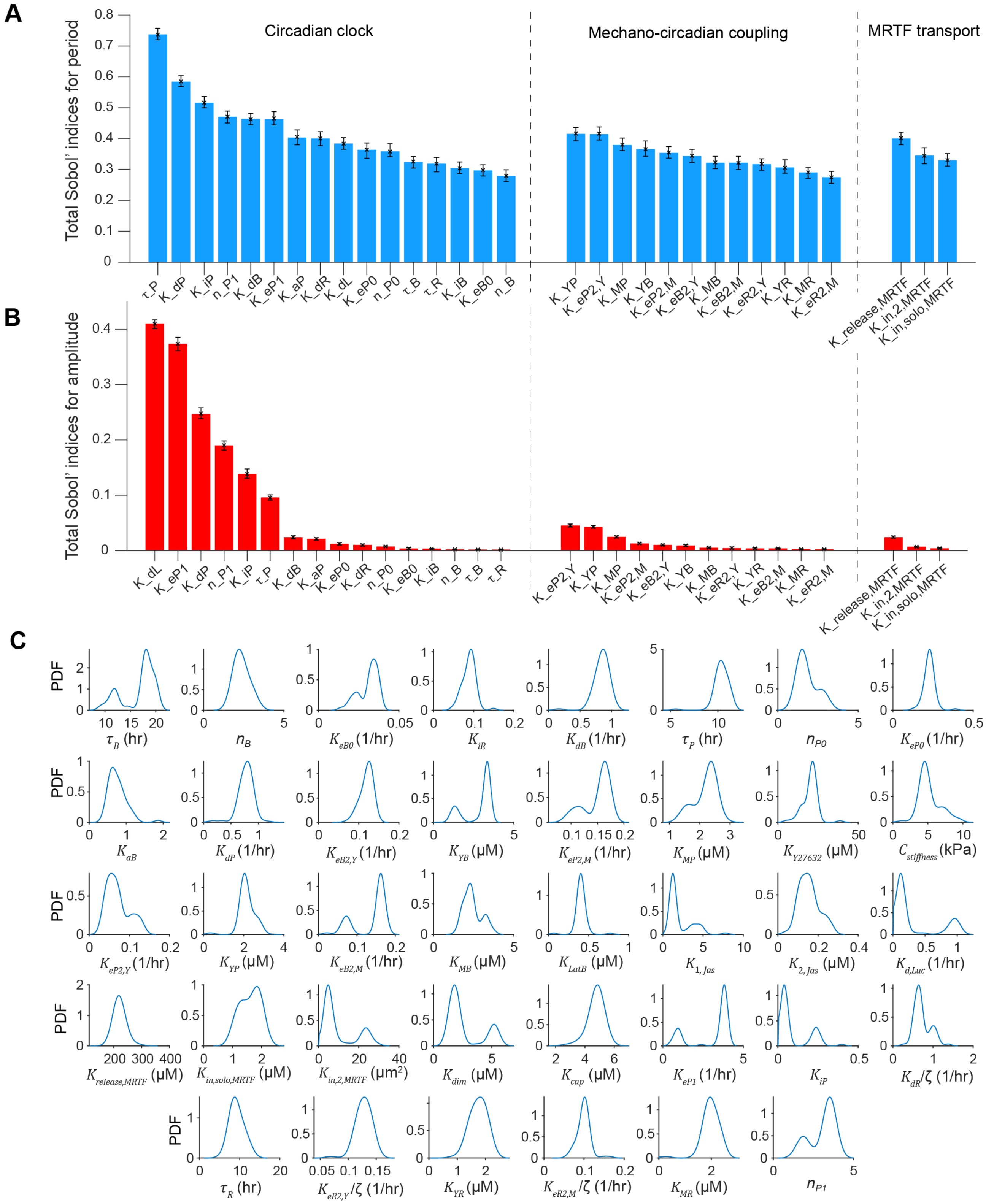

Journal of Cell Science • Supplementary information

Fig. S2. Sobol' sensitivity analysis and Bayesian parameter estimation. A-B) Total Sobol' indices associated with the luciferase oscillation period (A) and amplitude (B). Parameters are sorted into three categories: those related exclusively to the core circadian clock, those related to mechanotransduction-circadian coupling, and those governing MRTF transport. Within each category, parameters are plotted from the highest to the lowest estimated total Sobol' index. Markers with error bars denote the mean and 95% confidence intervals computed from bootstrapping with 100 replications. C) Parameter posterior distributions from Bayesian parameter estimation. Markov chain Monte Carlo (MCMC) was run for 1,000 iterations with 60 walkers. After discarding the first 500 iterations as burn-in, posterior distributions were estimated using kernel density estimation in MATLAB, with a bandwidth of 5% of the maximum value of a given parameter included in the prior distribution.

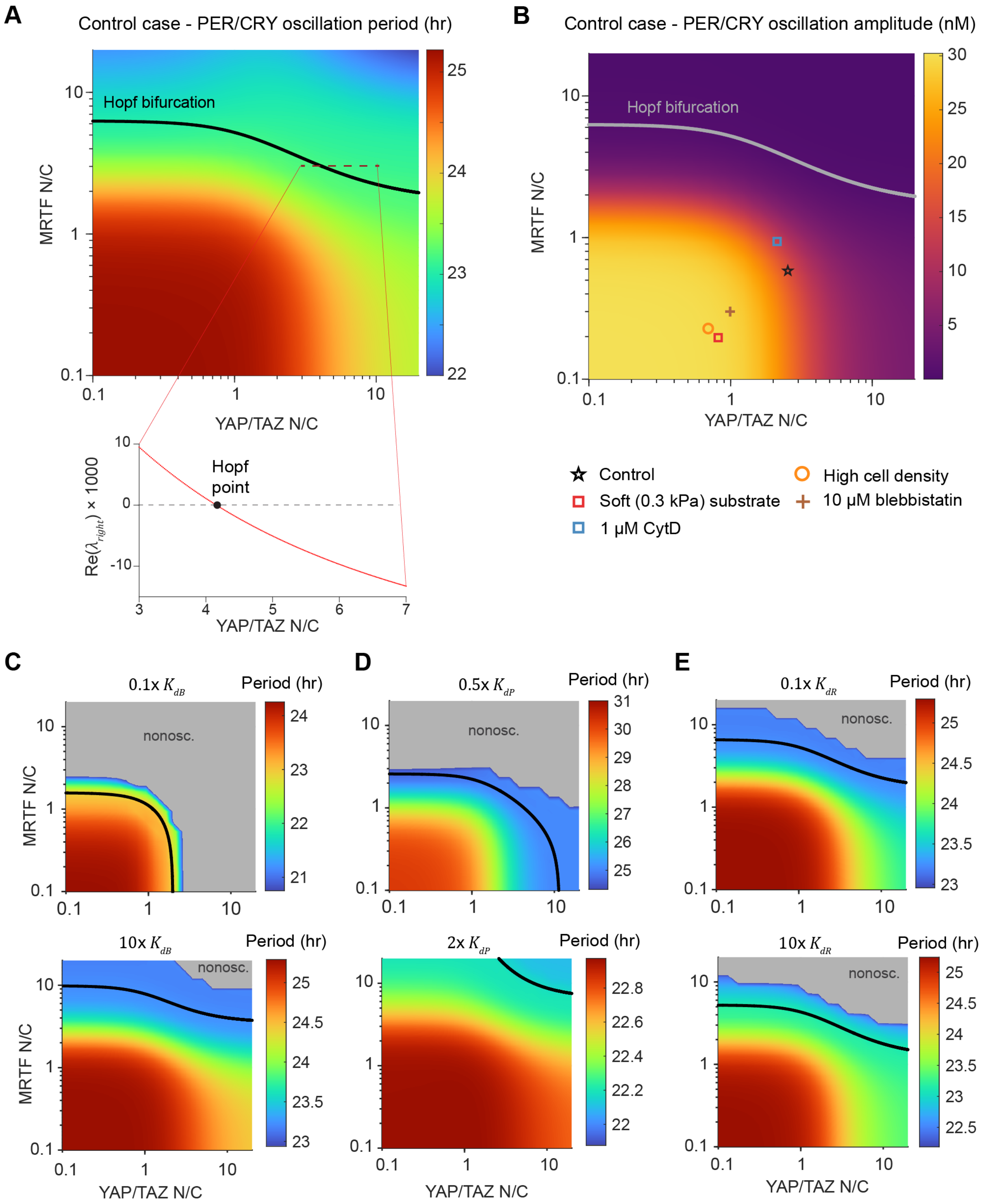

Journal of Cell Science • Supplementary information

Fig. S3. Linear stability analysis for different parameter values. A) Bifurcation diagram for control conditions, with oscillation period and location of the Hopf bifurcation plotted as a function of YAP/TAZ and MRTF nuclear to cytosolic ratios (N/C). The real part of the right-most pair of eigenvalues (λ_{right}) along the line where MRTF N/C is 3 (red dashed line in upper plot). The transition from positive to negative real part of the eigenvalues marks the occurrence of a Hopf bifurcation. B) Oscillation amplitude of PER/CRY over the MRTF-YAP/TAZ phase plane. Individual points correspond to tests run in Figure 5. C-E) Effects of decreasing or increasing K_{dB} (C), K_{dP} (D), or K_{dR} (E) on the location of the Hopf bifurcation and the oscillation period. The grey regions denote nonoscillatory solutions.

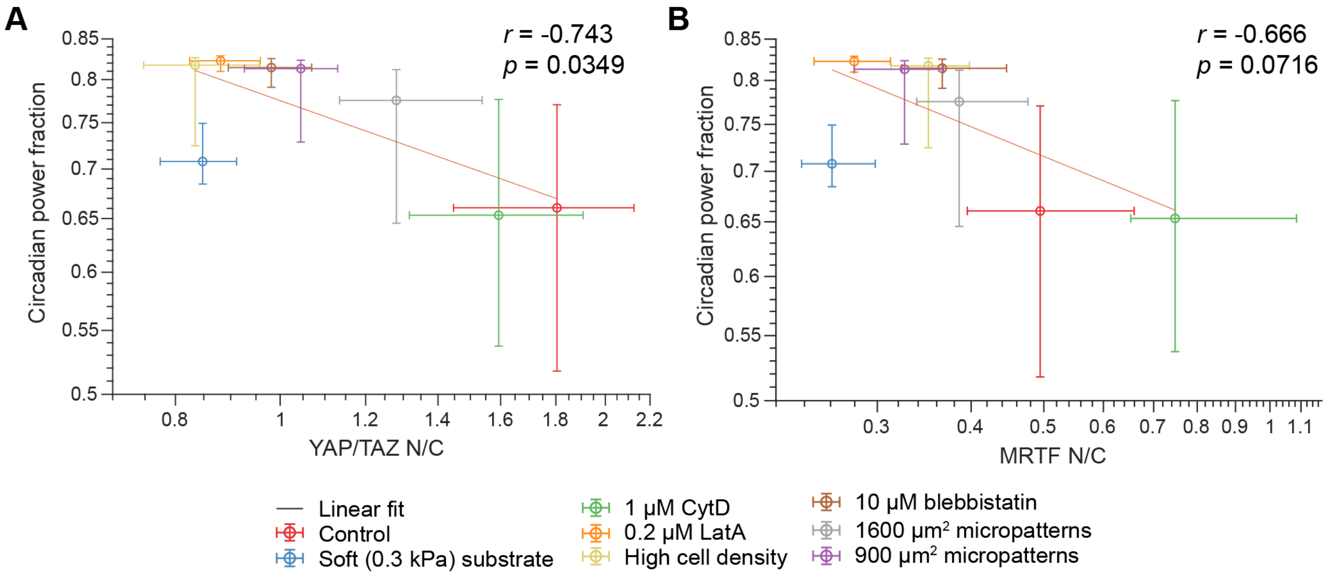

Fig. S4. Altered power fraction / transcriptional regulator correlation with increased expression of PER/CRY and BMAL1 on soft substrates. Increasing the baseline expression of PER/CRY and BMAL1 for cells on a soft substrate changes the correlations between circadian power fraction and YAP/TAZ N/C (A) or the MRTF N/C (B). In this case, we added additional baseline expression terms to Equations 9 and 10, each 0.0175 hr^{-1}. Pearson's correlation coefficient (between median YAP/TAZ or MRTF N/C and median circadian power fraction, both on the log scale), r, and the p-value associated with the null hypothesis, $r = 0$, are included on each graph. Data points denote median and error bars denote the range from the 40th to the 60th percentile for 200 model cells. To generate distinguishable colors for each condition, we used the linspecer tool in MATLAB (Lansey 2023).

Journal of Cell Science • Supplementary information

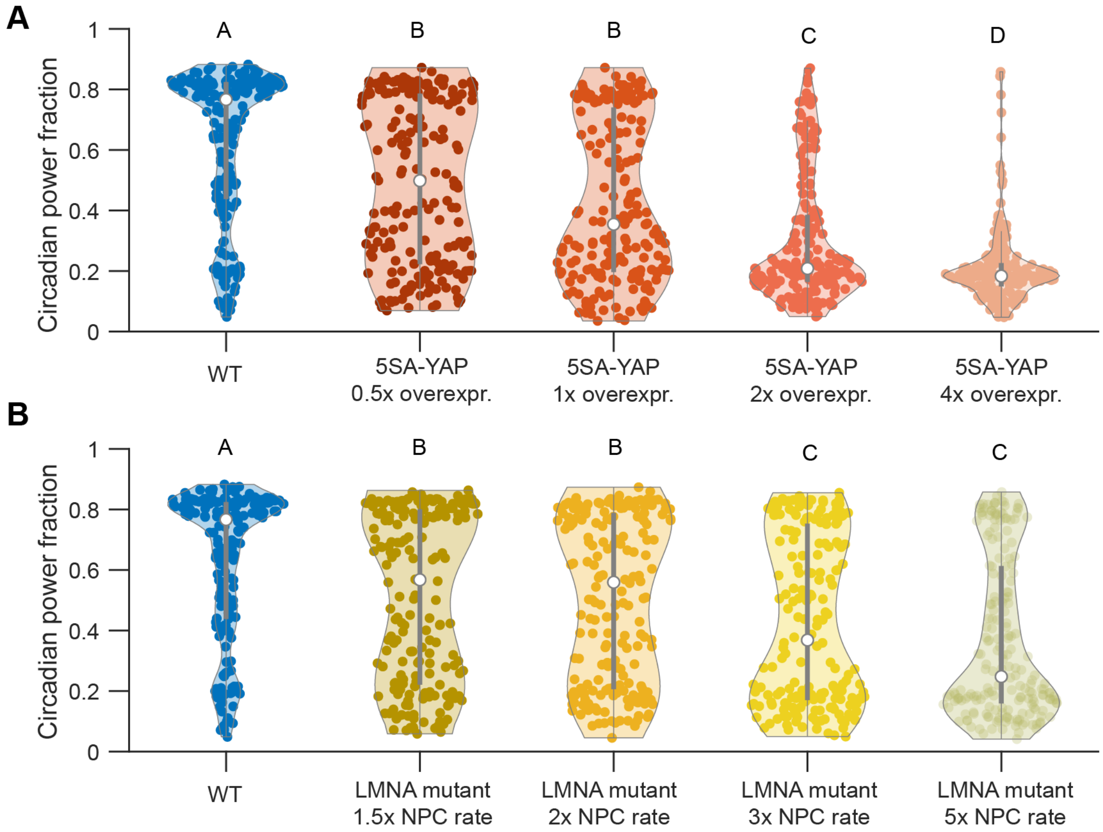

Fig. S5. Effects of alternative mutation parameters on model predictions. Circadian power fractions plotted for cells with different overexpression levels of YAP mutant (A) and different changes in NPC opening rate induced by LMNA mutation (B). 5SA-YAP overexpression is controlled by $K_{overexpress}$ and NPC opening rate is altered by changing K_{fNPC} (see Tables S2, S3 and S5). Compact letter display is used to denote statistical significance, where groups sharing a letter are statistically similar according to ANOVA followed by Tukey's *post hoc* test with a significance threshold of $p = 0.05$.

Journal of Cell Science • Supplementary information

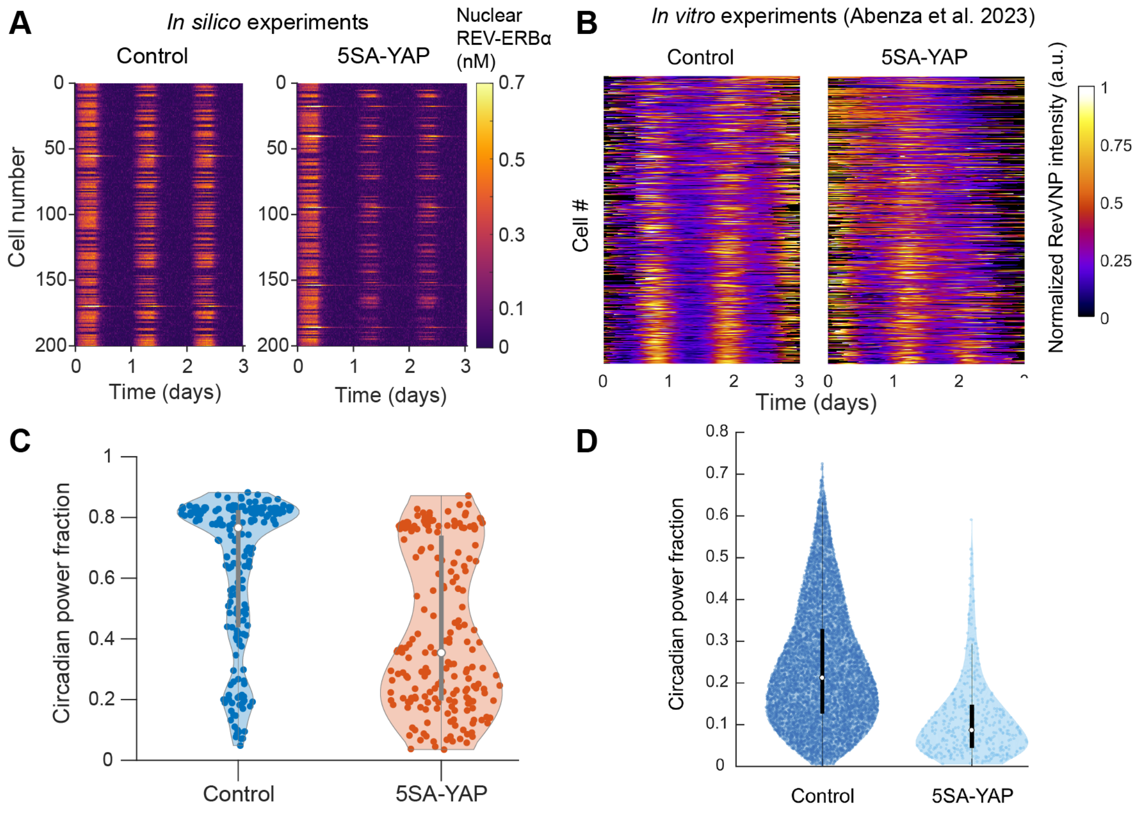

Fig. S6. Comparison of circadian oscillations for 5SA-YAP mutant in our study vs. Abenza et al. 2023 A) Kymographs depicting the dynamics of BMAL1 in *in silico* cell populations (wild type or 5SA-YAP mutants) on a 30 kPa substrate. Oscillations are substantially weaker for the population of mutant cells. B) Kymographs depicting the REV-VNP dynamics in *in vitro* cell populations (wild type or 5SA-YAP mutants) on a 30 kPa substrate. Oscillations are short-lived and/or inconsistent in the mutant population. C) Circadian power fraction for cells in each simulated population from panel A. 5SA-YAP mutants show a substantial reduction in the power fraction compared to control cells. D) Circadian power fraction for each population in the experiments shown in panel B. Circadian power fraction is much lower for 5SA-YAP mutant cells compared to control, similar to simulated populations. Panels B and D were published in *Journal of Cell Biology*; these plots were taken unaltered from Fig. 4 in Abenza et al. 2023 with the publisher's permission.

Table S1. Circadian clock model parameters.

Parameter	Units	Description	Baseline	Source	Fitting range (%)	MAP
τ_B	hr	BMAL1 delay constant	12.0	Tamaru et al. 2003	50-200	17.75
K_{eB0}	hr^{-1}	BMAL1-dependent BMAL1 expression rate	0.01	*	25-400	0.03564
K_{iR}	1	Inhibition constant for BMAL1 expression	0.04	Lema et al. 2000	25-400	0.08309
n_B	1	Hill coefficient for BMAL1 expression	2	Sriram et al. 2006	50-200	2.223
K_{dB}	hr^{-1}	BMAL1 degradation rate	0.4	Lema et al. 2000	25-400	0.9152
τ_P	hr	PER/CRY delay constant	7.5	Yagita et al. 2001<br>Yagita et al. 2002<br>Lee et al. 2001	50-200	10.59
K_{eP0}	hr^{-1}	BMAL1-dependent PER/CRY expression rate	0.1	*	25-400	0.2149
K_{aB}	1	Activation constant for PER/CRY expression	0.5	*	25-400	0.5068
n_{P0}	1	First Hill coefficient for PER/CRY expression	2	Lema et al. 2000	50-200	1.415
K_{eP1}	hr^{-1}	PER/CRY-dependent PER/CRY expression rate	1.0	*	25-400	3.835
K_{iP}	1	Inhibition constant for PER/CRY expression	0.1	*	25-400	0.02981
n_{P1}	1	Second Hill coefficient for PER/CRY expression	2	Sriram et al. 2006<br>Lema et al. 2000	50-200	3.362
K_{dP}	hr^{-1}	PER/CRY degradation rate	0.4	*	25-400	0.9478
τ_R	hr	REV-ERBα delay constant	7.5	Sriram et al. 2006	50-200	9.075
K_{dR}/ζ	hr^{-1}	REV-ERBα degradation rate (relative)	0.4	*	25-400	0.6888
$K_{eB2,Y}$	hr^{-1}	YAP/TAZ-regulated BMAL1 expression rate	0.05	*	25-400	0.1328
K_{YB}	nM	Activation constant for YAP/TAZ regulation of BMAL1 expression	1.0	*	25-400	3.109
$K_{eB2,M}$	hr^{-1}	MRTF-regulated BMAL1 expression rate	0.05	*	25-400	0.1609
K_{MB}	µM	Activation constant for MRTF regulation of BMAL1 expression	1.0	*	25-400	2.298
$K_{eP2,Y}$	hr^{-1}	YAP/TAZ-regulated PER/CRY expression rate	0.05	*	25-400	0.03885
K_{YP}	µM	Activation constant for YAP/TAZ regulation of PER/CRY expression	1.0	*	25-400	2.001
$K_{eP2,M}$	hr^{-1}	MRTF-regulated PER/CRY expression rate	0.05	*	25-400	0.1693
K_{MP}	µM	Activation constant for MRTF regulation of PER/CRY expression	1.0	*	25-400	2.309
$K_{eR2,Y}$	hr^{-1}	YAP/TAZ-regulated REV-ERBα expression rate	0.05	*	25-400	0.1356
K_{YR}	µM	Activation constant for	1.0	*	25-400	1.273

Continued on next page

Table S1. (continued.) Circadian clock model parameters.

Parameter	Units	Description	Baseline	Source	Fitting range (%)	MAP
		YAP/TAZ regulation of REV-ERBα expression				
$K_{eR2,M}$	hr^{-1}	MRTF-regulated REV-ERBα expression rate	0.05	*	25-400	0.1024
K_{MR}	µM	Activation constant for MRTF regulation of REV-ERBα expression	1.0	*	25-400	1.737
K_{dL}	hr^{-1}	Luciferase degradation rate	0.35	Feeney et al. 2016	25-400	0.1341
τ_L	hr	Luciferase delay constant	1	**	**	1
B_{ref}	nM	Reference BMAL1 concentration	190	†	†	190
P_{ref}	nM	Reference PER/CRY concentration	7.5	†	†	7.5
R_{ref}	nM	Reference REV-ERBα concentration	0.07	†	†	0.07

Fitting range specifies the range of the uniform prior distribution (in percentage) and "MAP" is the maximum *a posteriori* from Bayesian parameter estimation.

* Set based on early testing of the model, such that oscillations still occur when carrying over applicable parameter values from Lema et al. 2000.

** The choice of τ_L is arbitrary in this equation; it does not affect model behavior, as it simply sets the phase of luciferase oscillations, but changes in luceriferase do not feedback to affect any other dynamics.

† To convert dimensionless BMAL1, PER/CRY, and REV-ERBα concentrations to nM, we multiplied these concentrations by B_{ref}, P_{ref}, and R_{ref}, respectively. We set these parameters to match with experimental measurements (Smyllie et al. 2016) and previous models (Mirsky et al. 2009). These parameters are fixed and not used in fitting as our fitting protocol only examines relative changes in nuclear concentration (see Methods).

Table S2. Mechanotransduction model equations. The ODE expression and steady-state expression are given for each variable considered. Note that only the steady-state solutions were considered in this study.

Variable	Expression
$\frac{d[FAK_P]}{dt}$	$\left(k_f + k_{sf}\frac{E}{C_{stiffness}+E}\right)([FAK_{tot}] - [FAK_P]) - k_{df}[FAK_P]$
$[FAK_P]_{SS}$	$[FAK_{tot}]\frac{K_f(C_{stiffness}+E)+K_{sf}E}{(1+K_f)(C_{stiffness}+E)+K_{sf}E}$ where $K_f = \frac{k_f}{k_{df}}$, $K_{sf} = \frac{k_{sf}}{k_{df}}$
$\frac{d[RhoA_{GTP}]}{dt}$	$k_{fk\rho}(\gamma[FAK_P]^n+1)\left(\frac{[RhoA_{tot}]}{SA_{PM}} - [RhoA_{GTP}]\right) - k_{d\rho}[RhoA_{GTP}]$
$[RhoA_{GTP}]$	$\frac{[RhoA_{tot}]}{SA_{PM}}\frac{K_{fk\rho}(\gamma[FAK_P]^n_{SS}+1)}{1+K_{fk\rho}(\gamma[FAK_P]^n_{SS}+1)}$ where $K_{fk\rho} = \frac{k_{fk\rho}}{k_{d\rho}}$
$\frac{d[ROCK_A]}{dt}$	$k_{r\rho}[RhoA_{GTP}]\frac{([ROCK_{tot}]-[ROCK_A])SA_{PM}}{vol_{cyto}N_{convert}} - k_d[ROCK_A]$
$[ROCK_A]$	$[ROCK_{tot}]\frac{K_{r\rho}[RhoA_{GTP}]_{SS}SA_{PM}}{vol_{cyto}N_{convert}+K_{r\rho}[RhoA_{GTP}]_{SS}SA_{PM}}$, where $K_{r\rho} = \frac{k_{r\rho}}{k_d}$

Continued on next page

Table S2. (continued.) Mechanotransduction model equations.

Variable	Expressions
$\frac{d[mDia_A]}{dt}$	$k_{m\rho}[RhoA_{GTP}]\frac{([mDia_{tot}]-[mDia_A])SA_{PM}}{vol_{cyto}N_{convert}} - k_{dmDia}[mDia_A]$
$[mDia_A]_{SS}$	$[mDia_{tot}]\frac{K_{m\rho}[RhoA_{GTP}]_{SS}SA_{PM}}{vol_{cyto}N_{convert}+K_{m\rho}[RhoA_{GTP}]_{SS}SA_{PM}}$ where $K_{m\rho}=\frac{k_{m\rho}}{k_{dmDia}}$
$\frac{d[Myo_A]}{dt}$	$\frac{1}{2}k_{mr}\left(\epsilon[ROCK_A](\tanh\{s_1([ROCK_A]-[ROCK_{ref}])\}+1)+1\right)([Myo_{tot}]-[Myo_A])$ $-k_{dmy}[Myo_A]$
$[Myo_A]_{SS}$	$[Myo_{tot}]\frac{K_{mr}\left(\epsilon[ROCK_A]_{SS}(\tanh\{s_1([ROCK_A]_{SS}-[ROCK_{ref}])\}+1)+1\right)}{2+K_{mr}\left(\epsilon[ROCK_A]_{SS}(\tanh\{s_1([ROCK_A]_{SS}-[ROCK_{ref}])\}+1)+1\right)}$ where $K_{mr}=\frac{k_{mr}}{k_{dmy}}$
$\frac{d[LIMK_A]}{dt}$	$\frac{1}{2}k_{lr}\left(\tau[ROCK_A](\tanh\{s_1([ROCK_A]-[ROCK_{ref}])\}+1)+1\right)([LIMK_{tot}]-[LIMK_A])$ $-k_{dl}[LIMK_A]$
$[LIMK_A]_{SS}$	$[LIMK_{tot}]\frac{K_{lr}\left(\tau[ROCK_A]_{SS}(\tanh\{s_1([ROCK_A]_{SS}-[ROCK_{ref}])\}+1)+1\right)}{2+K_{lr}\left(\tau[ROCK_A]_{SS}(\tanh\{s_1([ROCK_A]_{SS}-[ROCK_{ref}])\}+1)+1\right)}$ where $K_{lr}=\frac{k_{lr}}{k_{dl}}$
$\frac{d[Cofilin_{NP}]}{dt}$	$k_{turnover}([Cofilin_{tot}]-[Cofilin_{NP}])-\frac{k_{cat,Cof}[LIMK_A][Cofilin_{NP}]}{k_{m,Cof}+[Cofilin_{NP}]}$
$[Cofilin_{NP}]_{SS}$	root $\left\{k_{turnover}([Cofilin_{tot}]-[Cofilin_{NP}]_{SS})-\frac{k_{cat,Cof}[LIMK_A]_{SS}[Cofilin_{NP}]_{SS}}{k_{m,Cof}+[Cofilin_{NP}]_{SS}}\right\}$
$\frac{d[F]}{dt}$	$\frac{1}{2}k_{ra}\left(\alpha[mDia_A](\tanh\{s_1([mDia_A]-[mDia_{ref}])\}+1)+1\right)([Actin_{tot}]-[F])$ $-(k_{dep}+k_{fc1}[Cofilin_{NP}])[F]$
$[F]_{SS}$	$\frac{[Actin_{tot}]K_{ra}\left(\alpha[mDia_A]_{SS}(\tanh\{s_1([mDia_A]_{SS}-[mDia_{ref}])\}+1)+1\right)}{2(1+K_{fc1}[Cofilin_{NP}]_{SS})+K_{ra}\left(\tau[mDia_A]_{SS}(\tanh\{s_1([mDia_A]_{SS}-[mDia_{ref}])\}+1)+1\right)}$ where $K_{ra}=\frac{k_{ra}}{k_{dep}}$, $K_{fc1}=\frac{k_{fc1}}{k_{dep}}$
$\frac{d[LaminA_{NP}]}{dt}$	$\frac{k_{fl}E_{cyto}}{C_{LaminA}+E_{cyto}}([LaminA_{tot}]-[LaminA_{NP}])-k_{rl}[LaminA_{NP}]$
$[LaminA_{NP}]_{SS}$	$[LaminA_{tot}]\frac{K_{fl}E_{cyto}}{C_{LaminA}+E_{cyto}(1+K_{fl})}$, where $E_{cyto}=p_{cyto}[F]^{2.6}$ and $K_{fl}=\frac{k_{fl}}{k_{rl}}$

Continued on next page

Journal of Cell Science • Supplementary information

Table S2. (continued.) Mechanotransduction model equations.

Variable	Expressions
$\frac{d[NPC_A]}{dt}$	$k_{fNPC}[LaminA_{NP}][F][MyoA]([NPC_{tot}]-[NPC_A])-k_r[NPC_A]$
$[NPC_A]_{SS}$	$[NPC_{tot}]\frac{K_{fNPC}[LaminA_{NP}]_{SS}[F]_{SS}[MyoA]_{SS}}{1+K_{fNPC}[LaminA_{NP}]_{SS}[F]_{SS}[MyoA]_{SS}}$ where $K_{fNPC}=\frac{k_{fNPC}}{k_r}$
$\frac{d[Y_{NP}]}{dt}$	$(k_{CN}+k_{CY}[F][Myo_A])([Y_{cyto}]-[Y_{NP}])-k_{NC}[Y_{NP}]-\frac{d[Y_{nuc}]}{dt}\frac{vol_{nuc}}{vol_{cyto}}$
$[Y_{NP}]_{SS}$	$[Y_{cyto}]\phi_{NP}$ where $[Y_{cyto}]=\frac{[Y_{tot}]-[Y_{nuc}]_{SS}vol_{nuc}N_{convert}}{vol_{cyto}N_{convert}}$, $K_{CN}=\frac{k_{CN}}{k_{NC}}$, $K_{CY}=\frac{k_{CY}}{k_{NC}}$, $\phi_{NP}=\frac{K_{CN}+K_{CY}[F]_{SS}[Myo_A]_{SS}}{K_{CN}+K_{CY}[F]_{SS}[Myo_A]_{SS}+1}$ *
$\frac{d[Y_{nuc}]}{dt}$	$\frac{SA_{NM}}{vol_{nuc}N_{convert}}((k_{inb}+k_{in}[NPC_A])[Y_{NP}]-k_{out}[Y_{nuc}])$
$[Y_{nuc}]_{SS}$	$\frac{[Y_{tot}]\phi_{NP}}{N_{convert}}\frac{K_{in,solo,Y}+K_{in,2,Y}[NPC_A]_{SS}}{(K_{in,solo,Y}+K_{in,2,Y}[NPC_A]_{SS})\phi_{NP}vol_{nuc}+vol_{cyto}}$ where $K_{inb}=\frac{k_{inb}}{k_{out}}$, $K_{in}=\frac{k_{in}}{k_{out}}$
$\frac{d[M_{nuc}]}{dt}$	$\frac{SA_{NM}}{vol_{nuc}N_{convert}}((k_{in,solo,MRTF}+k_{in,2,MRTF}[NPC_A])[M_{free}]-k_{out,MRTF}[M_{nuc}])$ where $[M_{free}]=[M_{cyto}]\frac{1}{1+\left(\frac{[G]}{K_{MRTF}}\right)^2}$ and $[G]=[Actin_{tot}]-[F]$
$[M_{nuc}]_{SS}$	$\frac{[M_{tot}]}{N_{convert}}\frac{K_{in,solo,MRTF}+K_{in,2,MRTF}[NPC_A]_{SS}}{(K_{in,solo,MRTF}+K_{in,2,MRTF}[NPC_A]_{SS})vol_{nuc}+\left(1+\left(\frac{[G]_{SS}}{K_{MRTF}}\right)^2\right)vol_{cyto}}$ where $K_{in,solo,MRTF}=\frac{k_{in,solo,MRTF}}{k_{out,MRTF}}$, $K_{in,2,MRTF}=\frac{k_{in,2,MRTF}}{k_{out,MRTF}}$, and $[G]_{SS}=[Actin_{tot}]-[F]_{SS}$

We include the rate of change and the associated steady-state solution for each variable in Scott et al. 2021. Note that we only use the steady-state relationships in our implementation here, which effectively eliminates one parameter in each equation. Therefore, only equilibrium constants (capital K's) are reported in Tables S3 and S4, while the lowercase k rate constants remain unused and unspecified. An "A" subscript denotes an activated form of a species, "P" denotes a phosphorylated form, and "NP" denotes a nonphosphorylated form. * ϕ_{NP} corresponds to the fraction of cytosolic YAP/TAZ that is not phosphorylated.

Table S3. YAP/TAZ mechanotransduction parameters

Parameter	Units	Description	Value
E	kPa	Substrate stiffness	(varies)
K_f	1	FAK basal activation constant	0.4286
K_{sf}	1	substrate-stiffness-dependent FAK activation constant	10.83
$K_{fk\rho}$	1	RhoA basal activation constant	0.02688
γ	μM^5	pFAK-dependent RhoA activation constant	77.56
n	1	Cooperativity factor between FAK and Rho	5
$K_{r\rho}$	μM^{-1}	RhoA-dependent ROCK activation constant	0.8100
$K_{m\rho}$	μM^{-1}	RhoA-dependent mDia activation constant	0.4000
K_{mr}	1	Myosin activation constant	0.4478
ϵ	μM^{-1}	ROCK-dependent amplification of myosin activation	36
s_1	μM^{-1}	Slope of $\tanh$ activation functions	20
$[ROCK_{ref}]$	μM	$[ROCK_A]$ threshold in activation functions	0.3
K_{lr}	1	LIMK activation constant	0.035
τ	μM^{-1}	ROCK-dependent amplification of LIMK activation	55.49
$k_{turnover}$	s^{-1}	Cofilin activation rate	0.04
$k_{cat,Cof}$	s^{-1}	Rate of catalysis for cofilin phosphorylation	0.34
$k_{m,Cof}$	μM	$[Cofilin_{NP}]$ at half max phosphorylation rate	4
K_{ra}	1	Actin polymerization constant	0.1143
α	μM^{-1}	mDia-dependent amplification of actin polymerization	50
$[mDia_{ref}]$	μM	$[mDia_A]$ threshold in activation functions	0.165
K_{fc1}	μM^{-1}	Cofilin-dependent deactivation of actin polymerization	1.1429
K_{fl}	1	Cytosolic stiffness-dependent lamin A activation constant	460
C_{laminA}	kPa	Threshold stiffness for lamin A activation	100
p_{cyto}	kPa-μM$^{-2.6}$	Proportionality between cytosolic stiffness and $[F]^{2.6}$	9×10^{-6}
K_{fNPC}	μm^{2}μM^{-1}	NPC opening constant	3.218×10^{-8}
K_{CN}	1	Baseline YAP/TAZ activation constant	4.0
K_{CY}	μM^{-2}	Stress-fiber-dependent YAP/TAZ activation constant	5.429×10^{-3}
$K_{in,solo,Y}$	1	Basal YAP/TAZ nuclear import constant	1
$K_{in,2,Y}$	μm^2	$[NPC_A]$-dependent YAP/TAZ nuclear import constant	10
vol_{cyto}	μm^3	Cytosolic volume	2300
SA_{PM}	μm^2	Plasma membrane surface area	1260
vol_{nuc}	μm^3	Nuclear volume	550
$[FAK_{tot}]$	μM	Total cytosolic $[FAK]$	1
$[RhoA_{tot}]$	molecules	Total RhoA molecules per cell	1.385×10^6
$[ROCK_{tot}]$	μM	Total cytosolic $[ROCK]$	1
$[mDia_{tot}]$	μM	Total cytosolic $[mDia]$	0.8
$[Myo_{tot}]$	μM	Total cytosolic $[Myo]$	5
$[LIMK_{tot}]$	μM	Total cytosolic $[LIMK]$	2
$[Cofilin_{tot}]$	μM	Total cytosolic $[Cofilin]$	2
$[Actin_{tot}]$	μM	Total cytosolic actin (G-actin monomers per volume)	500
$[LaminA_{tot}]$	#μm^{-2}	Total lamin A molecules in the nuclear membrane	3500
$[NPC_{tot}]$	#μm^{-2}	Total NPCs in the nuclear membrane	6.5
$[YAPTAZ_{tot}]$	molecules	Total YAP/TAZ molecules per cell	1.385×10^6
$[MRTF_{tot}]$	molecules	Total MRTF molecules per cell	1×10^6
$N_{convert}$	(#/μm^3)/μM	Unit conversion factor	602.2

Journal of Cell Science • Supplementary information

Table S4. Estimated MRTF mechanotransduction and inhibitor treatment parameters.

Parameter	Units	Description	Baseline	Source	Fitting range (%)	MAP
$K_{in,solo,MRTF}$	1	MRTF nuclear entry rate constant	1	Scott et al. 2021	25-400	1.132
$K_{in,2,MRTF}$	μm^2	Lamin-A-dependent MRTF nuclear entry rate constant	10	Scott et al. 2021	25-400	7.756
$K_{release,MRTF}$	μM	Binding affinity of MRTF to G-actin	100	*	25-400	219.8
$C_{stiffness}$	kPa	Threshold stiffness for FAK phosphorylation	3.25	Scott et al. 2021	25-400	4.450
K_{cap}	μM	Cytochalasin D binding affinity for F-actin capping	2	†	25-400	5.040
K_{dim}	μM	Cytochalasin D binding affinity for G-actin dimerization	2	†	25-400	1.756
K_{Y27632}	μM	Y27632 treatment EC50	10	†	25-400	22.12
K_L	μM	Latrunculin B treatment EC50	0.2	†	25-400	0.3947
$K_{1,Jas}$	1	Jasplakinolide treatment constant 1	2	Bubb et al. 2000 ‡	25-400	1.123
$K_{2,Jas}$	μM	Jasplakinolide treatment constant 2	0.1	Bubb et al. 2000 ‡	25-400	0.09520

Fitting range specifies the range of the uniform prior distribution (in percentage) and "MAP" is the maximum *a posteriori* from Bayesian parameter estimation.

* $K_{release,MRTF}$ initialized to a moderate value of G-actin based on the total amount of actin in the cell.

† Moderate treatment concentrations used in Xiong et al. 2022.

‡ Baseline values approximated from observed effects *in vitro*; see Fig 4 in Bubb et al. 2000.

Journal of Cell Science • Supplementary information

Table S5. Summary of different treatment effects.

Test	Treatment effect on parameters	$A_{contact}$ *
Cytochalasin D	$[F] \leftarrow$ Equation 22, $[G] \leftarrow$ Equation 23	5000
Latrunculin B	$K_{poly} \leftarrow \dfrac{K_{poly}}{1 + [LatB]/K_L}$	3000
Jasplakinolide	$K_{poly} \leftarrow K_{poly}\left(1 + (1 + K_{1,Jas})\dfrac{[J]}{K_{2,Jas}}\right)$	3000
Y27632	$K_{r\rho} \leftarrow \dfrac{K_{r\rho}}{1 + [Y27632]/K_{Y27632}}$	3000
High cell density	$K_{CN} \leftarrow \dfrac{K_{CN}}{K_{LATS}}$, $K_{CY} \leftarrow \dfrac{K_{CY}}{K_{LATS}}$ **	1200
Low cell density	(Abenza control)	3000
1600 µm^2 micropatterns	-	1600
900 µm^2 micropatterns	-	900
Latrunculin A	$K_{poly} \leftarrow \dfrac{K_{poly}}{1 + [LatA]/K_L}$ ***	600
Blebbistatin	$K_{CY} \leftarrow \dfrac{K_{CY}}{1 + [bleb]/K_{bleb}}$, $K_{fNPC} \leftarrow \dfrac{K_{fNPC}}{1 + [bleb]/K_{bleb}}$ †	4000
Soft substrate (Abenza)	$E = 0.3$kPa	1000
5SA-YAP mutant	$Y_{NP} \leftarrow Y_{NP} + Y_{NP}^{5SA}, Y_{nuc} \leftarrow Y_{nuc} + Y_{nuc}^{5SA}$ where Y_{NP}^{5SA} and Y_{nuc}^{5SA} are Y_{NP} and Y_{nuc} eval. for $K_{CN} \leftarrow \infty$, $K_{CY} \leftarrow \infty$, and $[YAPTAZ_{tot}] \leftarrow K_{overexpress}[YAPTAZ_{tot}]^{\ddagger}$	§
Lamin A mutant	$K_{fl} \leftarrow \infty$ $K_{fNPC} \leftarrow 2K_{fNPC}$	§

For population-level models, K_{dim}, K_{cap}, K_L, $K_{1,Jas}$, $K_{2,Jas}$, and K_{Y27632} were all sampled from their posterior distributions. K_{LATS}, K_{bleb}, $K_{overexpress}$, and $A_{contact}$ were sampled from log-normal distributions centered at their specified baseline values.

* Incorporated through Equations 31 and 32.

** K_{LATS} captures the increased phosphorylation of YAP/TAZ due to LATS activation; its baseline value is 3, to match the maximum LATS-mediated fold increase in YAP/TAZ phosphorylation rate in Sun et al. 2016

*** For simplicity, Latrunculin A is assumed to have the same treatment response as Latrunculin B.

† The baseline value for K_{bleb} is 1 µM.

‡ The baseline value for $K_{overexpress}$ is 1 (same expression level as native YAP/TAZ).

§ Mutant cells are assumed to adopt the same contact areas as wild type cells under a given condition.

Journal of Cell Science • Supplementary information

Table S6. Summary of calibration data.

Condition	Mean period	Std period	Mean ampl	Std ampl
Control (Tot) *	24.25	0.4239	1	0.3996
Control (glass)	23.84	0.1748	1	0.3739
300 kPa substrate	24.59	0.2330	2.295	0.1094
19 kPa substrate	25.59	0.3350	1.939	0.2553
Control (Y27632)	24.71	0.1659	1	0.0090
10 µM Y27632	25.10	0.2341	1.306	0.1081
20 µM Y27632	25.17	0.3024	1.153	0.1081
Control (CytD)	24.21	0.2060	1	0.1954
1 µM CytD	25.00	0.1803	1.287	0.1494
2 µM CytD	24.97	0.0258	1.655	0.3218
5 µM CytD	24.64	0.3991	1.943	0.3908
Control (LatB)	24.94	0.1048	1	0.0235
1 µM LatB	25.41	0.1921	1.612	0.1412
2 µM LatB	25.97	0.2620	2.377	0.0588
Control (Jas)	23.68	0.2487	1	0.1250
0.1 µM Jas	23.22	0.2902	0.953	0.1797
0.2 µM Jas	22.39	0.4145	1.094	0.2344
0.5 µM Jas	20.40	0.1658	0.2969	0.0703

* Weighted averages of control data (weighted by number of data points associated with each measurement). Standard deviation was computed as the root mean weighted sum of squares (the square root of the mean variance).
