# Supplementary material for: Computational modeling establishes mechanotransduction as a potent modulator of the mammalian circadian clock
Source: J Cell Sci. 2024 Sep 9;137(17):jcs261782. doi: 10.1242/jcs.261782 (PMC11423814; doi:10.1242/jcs.261782)
Supplement: Supplementary file 1 [file joces-137-261782_review_history.pdf]

Journal of Cell Science

Computational modeling establishes mechanotransduction as a potent modulator of the mammalian circadian clock

Emmet A. Francis and Padmini Rangamani
DOI: 10.1242/jcs.261782

Editor: Megan King

Review timeline
Original submission: 3 January 2024
Editorial decision: 4 March 2024
First revision received: 18 June 2024
Accepted: 5 August 2024

Original submission

First decision letter

MS ID#: JOCES/2023/261782

MS TITLE: Computational modeling establishes mechanotransduction as a potent entrainment cue for the mammalian circadian clock

AUTHORS: Emmet A Francis and Padmini Rangamani

We have now reached a decision on the above manuscript.

To see the reviewers' reports and a copy of this decision letter, please go to: https://submit-jcs.biologists.org and click on the 'Manuscripts with Decisions' queue in the Author Area. (Corresponding author only has access to reviews.)

As you will see, the reviewers raise a range of criticisms from minor to substantial that prevent me from accepting the paper in its current form. They suggest, however, that a revised version might prove acceptable, if you can address their concerns. If you think that you can deal satisfactorily with the criticisms on revision, I would be pleased to see a revised manuscript. We would then return it to the reviewers.

Please ensure that you clearly highlight all changes made in the revised manuscript. Please avoid using 'Tracked changes' in Word files as these are lost in PDF conversion.

I should be grateful if you would also provide a point-by-point response detailing how you have dealt with the points raised by the reviewers in the 'Response to Reviewers' box. Please attend to all of the reviewers' comments. If you do not agree with any of their criticisms or suggestions please explain clearly why this is so.

Reviewer 1

Advance summary and potential significance to field

In this study the authors coupled a mechanoregulatory model to a model of the circadian clock. They show that YAP/TAZ and MRTF nuclear accumulation influences the amplitude and frequency of circadian genes/proteins, corresponding to experimental literature. They also show that

© 2024. Published by The Company of Biologists under the terms of the Creative Commons Attribution License (https://creativecommons.org/licenses/by/4.0/).

YAP/TAZ nuclear accumulation seems to play a more influential role than MRTF and provide testable predictions to rescue the circadian rhythm in cells with particular mutations. I highly compliment the authors on the extensive and rigorous work, which I find very exciting and of great importance to the field.

Comments for the author

The manuscript is well structured, I only have minor concerns, mainly to improve readability and reproducibility.

Recent experimental studies have revealed connections between disease states and disrupted circadian rhythms in different cell types [6, 7].

Might be nice to work out more as this can enhance the impact of the work; which other proteins might be interesting to simulate with this framework?

… resulting expressions for each steady-state quantity are given in Table S1.

Consider adding the full system of equations to the supplement, just for reproducibility and to allow checking the steady state calculations

I assume nr is related to SApm?

In the PNAS equation there is kinb, kin, kout for YAPTAZnuc – here it is kin_solo kin_2_y; Check for consistency.

Line 119 – "we develop a minimal model"
How do you know it is minimal?

"The relationships used in our model are primarily motivated by the single DDE model used in [20] and the more detailed ODE model used in [19]."
Why? Considering there is an extensive body of literature, it might be good to elaborate the motivation of your choice – why are these the best models?

We note that this self-inhibition is indirect in reality, ultimately mediated by the nuclear receptor, REV-ERBα [3].
I think it is important to elaborate this a bit – what is this indirect mechanism? Also to clarify why modeling it with a delay is then appropriate.

Eq 1-2- why this formalism? Why order 2?
Same for equations 3& 4 ?

Line 162 – "to all free parameters in our model (Figure S1A)." It is not clear what the free parameters are. Consider highlighting this more.
Was the Bayesian calibration done for all parameters/perturbations at the same time or separately? If so, how?

Table S4 – the mutant cases could benefit from some extra explanation; why these choices – on which experimental data are they based?

We note that although our model does not capture the full dramatic decrease in oscillation period upon jasplakinolide treatment in [14].
Can you explain (from the mathematical model) why not? Should this fit not be better in order to proceed with further simulations?

Very nice to try to capture the population level variability! These distributions were either assumed to be log-normal or were directly adopted from the posterior distributions derived from Bayesian parameter estimation (Section "Simulation of cell populations", 230 Figure S2) to represent a range of statistically likely parameter sets.
Furthermore, to capture additional sources of measurement noise, we added Gaussian white noise to each time series after solving the DDEs.

Later I understood that the posterior distributions are for the parameters linking the circadian model & mechanoregulatory model and the lognormal for the YAP/TAZ model. It would help readability to repeat this here.

© 2024. Published by The Company of Biologists under the terms of the Creative Commons Attribution License (https://creativecommons.org/licenses/by/4.0/).

What is the substantiation for the log-normal? How does this correspond to the real distribution?

Nuclear abundance of YAP/TAZ and MRTF correlate with the strength of circadian oscillations across treatment conditions

I am confused about this section as many of these datasets were used to calibrate the model; so which new aspects do you learn from it now?

This agrees with our findings in Figure S5, where the stability of oscillations often depends most strongly on the amount of nuclear YAP/TAZ.

Can this be explained mathematically? Why (in the model/signaling network) is YAP/TAZ more important for the oscillations? How is this dependent on the parameter settings?

It is not clear how figure S6 is calculated/obtained.

"… model by introducing an equal amount of mutant 5SA-YAP as native YAP/TAZ"
And keeping the total amount of YAP constant? (so replacing half of the native by mutant)
Or increasing the total amount of YAP by half?

For the mutations, did you also explore other settings, e.g. various opening rate of NPCs and various mutant 5SA-YAP levels (not equal amount)? How does that influence your conclusions?

Typos:
- Line 29 - REV-ERBα – also write in full?
- that mutated the lamin 300 A was constitutively active -> that the mutated lamin A… (typo)

Reviewer 2

Advance summary and potential significance to field

This in silico modelling paper by Francis and Rangamani is a very interesting read. It is very well written. It builds on recent experimental observations for mechanical control of cell intrinsic clocks, previous modelling of clocks and mechanical pathways. There are several new directions predicted that can be experimentally validated in future, such as the mechanical rescue of disrupted rhythms due to mutations in YAP or Lamin. The finding that YAP is more dominant than MRTF in regulating clocks is also new and relevant. These findings will shed light on this emerging new model of regulation of circadian clocks by tissue mechanics, which could have implications in cancer, fibrosis or laminopathies.
However, there are significant flaws in the modelling parameters, which prevented enthusiasm and must be addressed before further consideration.

Comments for the author

- The use of nuclear BMAL1 to measure clock dynamics is unfortunate. It has been shown by quantitative imaging and math models that BMAL1 proteins hardly oscillate in the nucleus with a very small amplitude, which is very different from what is being proposed in the modelling here (e.g. Fig.4 and Fig.1B/C). It is well known that the negative arm of the clock is the more rhythmic arm. Please see PMID: 19549830; PMID: 32352975
- This paper has not taken into account cell-type dependent responses of clocks to mechanical cues, which as been reported before at least for epithelial cells and fibroblasts. The paper was cited but not properly discussed, nor modelled.
- It is unclear how cells on glass culture, which the authors mentioned to be extremely stiff (10 GPa), can be used for amplitude normalisation in Figure 2.
- There is a misunderstanding of the term entrainment, which is different from clock resetting, synchronisation, or modulation. To qualify for an entrainment time cue, the signal got to be rhythmic which then entrain the phase of the oscillation. As such, the word "entrainment cue" in the title and throughout the paper should be replaced, for example, with "Regulator" or "modulator".

© 2024. Published by The Company of Biologists under the terms of the Creative Commons Attribution License (https://creativecommons.org/licenses/by/4.0/).

First revision

Author response to reviewers' comments

We would like to sincerely thank both reviewers for their thoughtful feedback and attentive detail to the manuscript. We have made several substantial revisions to the manuscript according to points raised by each reviewer, including a major modification of our model's mathematical formulation. The main findings of this paper remain unchanged, but the model is significantly stronger and provides a better match with experiments, especially with respect to the magnitude of BMAL1 oscillations mentioned by Reviewer 2. This addition to the model is detailed in full below; in short, it involved introducing an additional delay-regulated expression term and considering the dynamics of REV-ERBα in addition to BMAL1 and PER/CRY.

Please note that the relevant changes in the manuscript and some relevant figures are included here for convenience, and changes in the revised text's PDF are denoted in blue.

Reviewer 1

Advance Summary and Potential Significance to Field:
In this study, the authors coupled a mechanoregulatory model to a model of the circadian clock. They show that YAP/TAZ and MRTF nuclear accumulation influences the amplitude and frequency of circadian genes/proteins, corresponding to experimental literature. They also show that YAP/TAZ nuclear accumulation seems to play a more influential role than MRTF and provide testable predictions to rescue the circadian rhythm in cells with particular mutations. I highly compliment the authors on the extensive and rigorous work, which I find very exciting and of great importance to the field.

We thank the reviewer for their enthusiastic evaluation of our work and for the detailed thoughtful feedback, particularly with respect to significance to the field. Below, we respond to each of the points raised.

Reviewer 1 Comments for the Author:
The manuscript is well structured, I only have minor concerns, mainly to improve readability and reproducibility.

1. *"Recent experimental studies have revealed connections between disease states and disrupted circadian rhythms in different cell types [6, 7]". Might be nice to work out more as this can enhance the impact of the work; which other proteins might be interesting to simulate with this framework?*
   In our new formulation of the model, we consider REV-ERBα as an additional component of the circadian network. There remain many other molecular details that have been examined in other models, such as RORc dynamics and separate isoforms of PER and CRY (PER1, PER2, CRY1, and CRY2) (Leloup and Goldbeter 2003; Mirsky et al. 2009). Our goal here was to include a minimal number of model components while still being able to leverage this work against recent experimental data. That said, we recognize the need to consider additional biological complexity in the future, which we now mention in lines 993-998: "These mechanosensitive effects could well extend to other circadian proteins such as RORc and may have different impacts on different isoforms of PER and CRY. Such details are neglected in the minimal modeling approach we adopt here, but could be well-addressed by integrating future experimental results with more detailed models."
2. *". . . resulting expressions for each steady-state quantity are given in Table S1". Consider adding the full system of equations to the supplement, just for reproducibility and to allow checking the steady state calculations.*

   Thank you for this suggestion, we have now added the full set of equations in Table S2.

3. *I assume nr is related to SApm?*
   The factor n_r from Scott et al. (Scott et al. 2021) was a fixed geometrical factor specifying the cytosolic volume to plasma membrane surface area in a reference cell geometry. We use the same values of surface area and volume in our model as in Scott et al., but we

© 2024. Published by The Company of Biologists under the terms of the Creative Commons Attribution License (https://creativecommons.org/licenses/by/4.0/).

split them into SA_{PM} and vol_{cyto} rather than including them in a single factor.

4. *In the PNAS equation there is kinb, kin, kout for YAPTAZnuc – here it is kinsolo kin2y; Check for consistency.*
   Thank you for pointing this out, we have changed the notation to be consistent with the Scott et al. paper.
5. *Line 119 – "we develop a minimal model" How do you know it is minimal?*
   This is an important point worth expanding on further. In short, our approach was to develop a model that is as simple as possible, but no simpler, to represent the circadian oscillations and mechanotransduction- circadian coupling. We acknowledge that often times, modeler preferences dictate modeling choices.
   Briefly, we justify our choice of model complexity as follows: while a simpler model of circadian oscillation is possible (e.g., a single DDE, as in [20]), this would not be capable of capturing both the positive and negative arms of the circadian clock that are essential to this study. We note that the original version of our model does leave out one important feature, that is, the negative regulation of BMAL1 expression by REV-ERBα, instead relying on a direct mechanism for BMAL1 self-inhibition. We have now included this extra detail in a new iteration of our model. Our results are consistent with the original findings and the model better matches the biological understanding. Furthermore, we have revised the wording on line 202; rather than referring to our model as minimal, we more accurately call it "reduced-order".
6. *"The relationships used in our model are primarily motivated by the single DDE model used in [20] and the more detailed ODE model used in [19]." Why? Considering there is an extensive body of literature, it might be good to elaborate the motivation of your choice – why are these the best models?*
   We chose these two studies as two ends of the spectrum, from the simplest possible circadian model to a more comprehensive model of circadian regulation. We have now referenced additional studies in our model development to demonstrate our consideration of other model features (lines 208-211): "This reduced approach is made possible by using DDEs, as in multiple previous circadian models of varying complexity (Lema et al. 2000; Sriram et al. 2006; Smolen et al. 2001). In addition to these DDE models, we integrate knowledge from previous ODE-based models of the mammalian circadian clock (Leloup and Goldbeter 2003; Mirsky et al. 2009; Forger and Peskin 2003)."
7. *"We note that this self-inhibition is indirect in reality, ultimately mediated by the nuclear receptor, REV- ERBa [3]". I think it is important to elaborate this a bit – what is this indirect mechanism? Also to clarify why modeling it with a delay is then appropriate.*
   Thank you for this comment, we certainly agree that this is an important model feature not given enough consideration in our original manuscript. Our new iteration of the model explicitly considers REV-ERBα as a separate variable (see note 19 to Reviewer 2 below).
8. *Eq 1-2- why this formalism? Why order 2? Same for equations 3 & 4 ?*
   The choice of Hill coefficient in such DDE models has previously been demonstrated to have a significant effect in simple models of circadian oscillations (Lema et al. 2000). In (Lema et al. 2000), a Hill coefficient of 2.5 was used, but they noted that oscillations were sustained for Hill coefficients greater than 1.39, justifying our initial choice of n = 2. However, given the importance of this coefficient, we included such Hill coefficients as free parameters in our new fit and sensitivity analysis (Table S1, Fig S2). In particular, we fit three separate Hill coefficients for REV-ERBα-mediated inhibition of BMAL1 expression (n_B in Eq 1), BMAL1-mediated activation of PER/CRY and REV-ERBα expression (n_{P0} in Eq 2), and PER/CRY-mediated inhibition of PER/CRY and REV-ERBα expression (n_{P1} in Eq 3).
   Separate testing shows that the Hill coefficient in the equations coupling circadian protein expression to YAP/TAZ and MRTF are of less fundamental importance to the model. These simply affect the steepness of the transition in oscillation period and/or Hopf bifurcation. This is illustrated in Figure S1 with an additional note in on lines 386-388: "For each of these expression functions in Equations 6 to 8, the Hill coefficient was fixed at 2 as it was not found to affect the qualitative behavior of our model (Figure S1)".

© 2024. Published by The Company of Biologists under the terms of the Creative Commons Attribution License (https://creativecommons.org/licenses/by/4.0/).

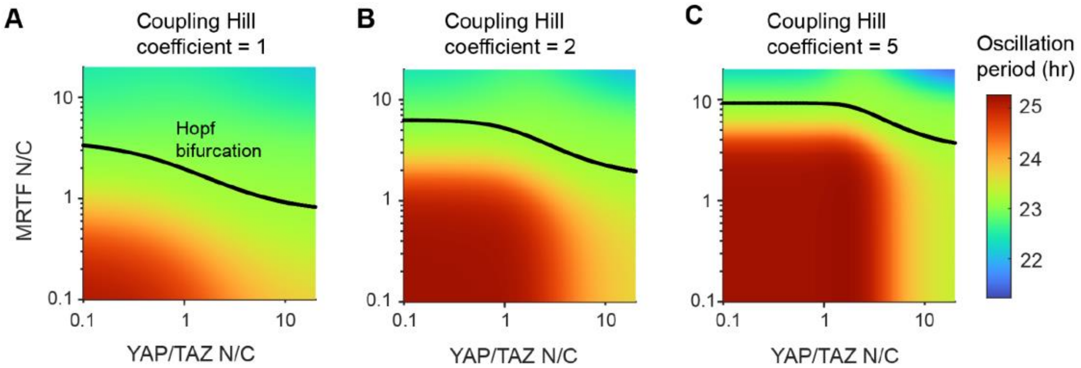

Effect of Hill coefficient on mechanotransduction-circadian coupling. Oscillation period and Hopf bifurcation plotted over the MRTF-YAP/TAZ phase plane for different choices of Hill coefficient in Equations 6-8. For simplicity, the same Hill coefficient was used in all equations for each case, either 1 (A), 2 (B, same as all other tests in the paper), or 5 (C). Changing the Hill coefficient simply shifts the Hopf bifurcation line and oscillation period without changing the qualitative behavior of the system.

9. *Line 162 – "to all free parameters in our model (Figure S1A)." It is not clear what the free parameters are. Consider highlighting this more. Was the Bayesian calibration done for all parameters/perturbations at the same time or separately? If so, how?*
   Thank you for this clarifying question. The free parameters were those in Tables S1 and S4, except those explictly noted as fixed. All parameters relevant to YAP/TAZ mechanotransduction except substrate stiffness sensitivity were fixed in the Bayesian calibration. We have clarified this in lines 427-431: "Accordingly, we estimated all free parameters, including circadian parameters, MRTF transport parameters, and inhibitor treatment-related parameters, rather than fix any at arbitrary default values. YAP/TAZ-related parameters were fixed to the values previously estimated by Scott et al. (Scott et al 2021)." The fit was done for all of these parameters at the same time as described in Methods.
10. *Table S4 – the mutant cases could benefit from some extra explanation; why these choices – on which experimental data are they based?*
   The 5SA-YAP mutant is based on the measurements in Abenza et al. (Abenza et al. 2023), in the limit of zero phosphorylation. The Lamin A mutant is postulated here, based lamin-related defects characterized in previous experimental work (Torvaldson et al. 2015; Cenni 2005; Mitsuhashi et al. 2010; Swift et al. 2013; Dutta et al. 2018; Owens et al. 2020), but we emphasize that this effect is postulated and not meant to accurately reflect any particular experiment. We clarify this point in lines 890-895: "Although the effects of such a mutation were informed by the above studies, this condition is not meant to directly represent any one given lamin A mutation, but rather to predict the effects of some possible lamin-related defects."
11. *"We note that although our model does not capture the full dramatic decrease in oscillation period upon jasplakinolide treatment in [14]." Can you explain (from the mathematical model) why not? Should this fit not be better in order to proceed with further simulations?*
   We agree that this is a potential concern. There is no fundamental mathematical reason that the decrease in oscillation period could not be captured, but our original objective function did not sufficiently penalize deviations from experimentally measured oscillation periods. Therefore, we decided to augment our objective function, adding an extra penalty to any parameter sets not matching the experimental measurements of oscillation period. As shown in the new model fit, this results in trends matching the experimental measurements in all cases.

© 2024. Published by The Company of Biologists under the terms of the Creative Commons Attribution License (https://creativecommons.org/licenses/by/4.0/).

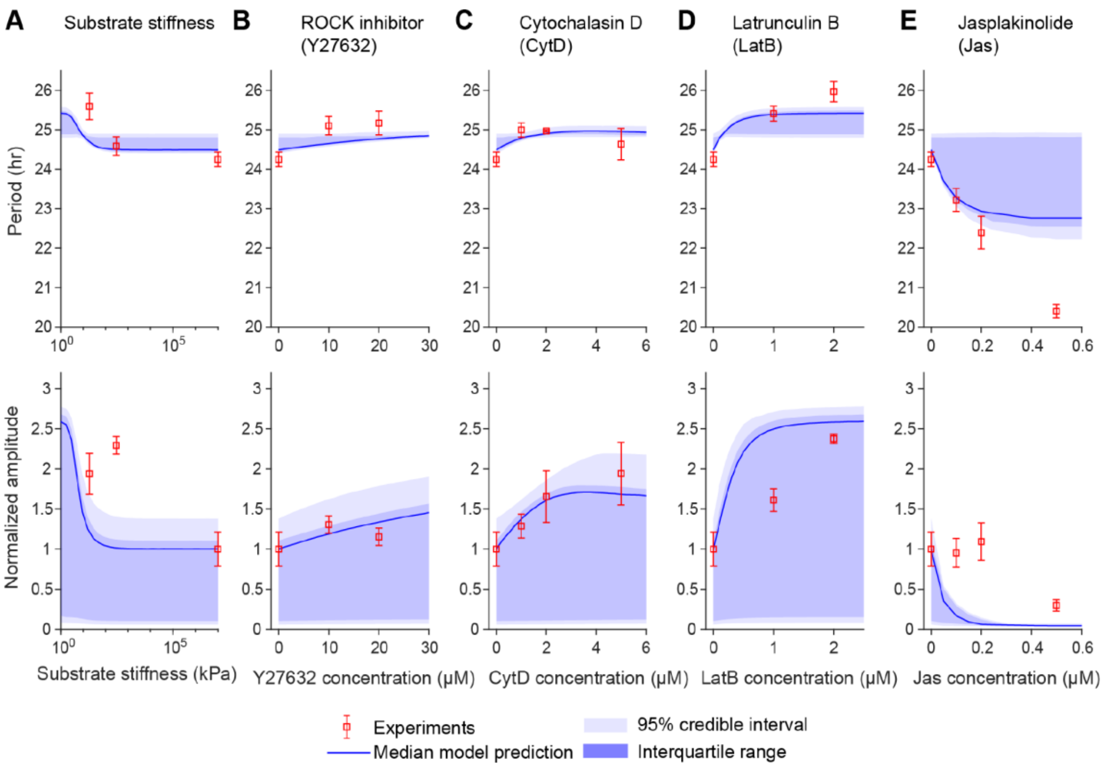

Bayesian parameter estimation for mechanotransduction-circadian model. A-E) Predicted oscillation period and amplitude in 5 experiments from Bayesian parameter estimation. Experimental data from Xiong *et al.* (Xiong et al. 2022) and model values are plotted for the effects of substrate stiffness (A), ROCK inhibitor (Y27632) treatment (B), cytochalasin D treatment (C), latrunculin B treatment (D), and jasplakinolide treatment (E). Experimental error bars denote standard deviation. For the model, the darker blue region indicates interquartile range and the lighter blue region spans the 95% credible interval (region from the 2.5 percentile to 97.5 percentile), based on sampling from the posterior distributions for parameter values. 1000 samples were generated to compute the estimates in this figure. In all cases, amplitude is normalized to that associated with the control condition (untreated cells on glass).

The changes to the objective function are detailed in the newly expanded methods section, “Sensitivity analysis and parameter estimation” (see equations 13-16).

12. *Very nice to try to capture the population level variability! “These distributions were either assumed to be log-normal or were directly adopted from the posterior distributions derived from Bayesian parameter estimation (Section “Simulation of cell populations”, Figure S2C) to represent a range of statistically likely parameter sets. Furthermore, to capture additional sources of measurement noise, we added Gaussian white noise to each time series after solving the DDEs.” Later I understood that the posterior distributions are for the parameters linking the circadian model & mechanoregulatory model and the lognormal for the YAP/TAZ model. It would help readability to repeat this here. What is the substantiation for the log-normal? How does this correspond to the real distribution?*

    Thank you for the suggestion, we have revised the statement as follows (lines 585-586): “These distributions were directly adopted from the posterior distributions derived from Bayesian parameter estimation or, in the case of fixed parameters in the YAP/TAZ model, were assumed to be log-normal.” We chose a log-normal distribution as biological variables commonly follow such a distribution (Koch 1966; Zhang and Popp 1994). This comes down to the errors in biological systems often being multiplicative rather than additive. Log-normal distributions have been adopted to introduce variability in biological networks elsewhere (Ni et al. 2018), and they have the added benefit of only supporting positive values of a given variable. The true distributions for such rate constants are generally not known, but we do note that the derived distributions for other variables in our Bayesian parameter estimation are often long-tailed themselves.

© 2024. Published by The Company of Biologists under the terms of the Creative Commons Attribution License (https://creativecommons.org/licenses/by/4.0/).

13. *Nuclear abundance of YAP/TAZ and MRTF correlate with the strength of circadian oscillations across treatment conditions I am confused about this section as many of these datasets were used to calibrate the model; so which new aspects do you learn from it now?*
Thank you for this clarifying question. First, we note that the data included in this figure directly were not used in calibration (although some of the conditions do indeed carry over). All of this data was from Abenza et al. (Abenza et al. 2023), whereas our calibration only used data from Xiong et al. (Xiong et al. 2022). Furthermore, while we tested these treatments in Figures 2-3, we did not have a quantification of the "strength" of circadian oscillations across a cell population, as represented by the circadian power fraction here. We emphasize both of these points in revised lines 632-636: "While several of these treatments were considered in our model calibration and initial testing, here we directly leverage the model against this separate set of experiments and we mainly focus on the strength of oscillations at the cell population level."
14. *"This agrees with our findings in Figure S5, where the stability of oscillations often depends most strongly on the amount of nuclear YAP/TAZ." Can this be explained mathematically? Why (in the model/signaling network) is YAP/TAZ more important for the oscillations? How is this dependent on the parameter settings?*
This trend depends sensitively on the magnitude of the coupling parameters (e.g. for PER/CRY, $K_{eB2,Y}$ and $K_{Y\,B}$ vs. $K_{eB2,M}$ and K_{MB}. In the original fit, the YAP/TAZ coupling was stronger than MRTF, but this does not carry over to our new calibrated model. Rather, which regulator dominates clearly depends on where you are in the phase plane (see Fig 3). We have changed wording throughout to reflect this. However, it remains true that YAP/TAZ correlates more strongly with the circadian power fraction compared to MRTF. This can be understood by looking at the amplitude of oscillations over the region of the YAP/TAZ-MRTF phase plane tested in Figure 5. This amplitude plot is shown in the newly revised Fig S3B, also included below.
For baseline parameters, YAP/TAZ is clearly a stronger regulator of the strength of oscillations within this region. We have clarified this point starting on line 667: "This higher sensitivity of circadian oscillation amplitude to changes in YAP/TAZ is consistent with the fact that, among mechanotransduction- circadian coupling parameters, circadian oscillation amplitude is most sensitive to $K_{eP\,2,0}$ (Figure S2). Furthermore, we can clearly see that oscillation amplitude changes more quickly with YAP/TAZ over the YAP/TAZ-MRTF phase plane over the region corresponding to the conditions tested here (Figure S3B)".
15. *It is not clear how figure S6 is calculated/obtained.*
We apologize for the confusion on this point. We have prepared a revised version of this figure
(now Figure S4, also shown below) with an explanation that we hope is clearer. Rather than artificially shifting the power fraction values associated with a softer substrate, we tested the effects of increasing the baseline expression of BMAL1 and PER/CRY on soft substrates. To accomplish this, we added small expression terms K_{eB3} and K_{eP3} to equations 6-7 for cells on a soft substrate. In this case, the correlation between circadian power fraction and YAP/TAZ is preserved, but the correlation with MRTF is no longer significant. This is clarified, starting on line 660:
"Additionally, we noted that cells exhibited low circadian power fractions even on soft substrates in Abenza et al. 2023. Accordingly, we postulated that an additional mechanism might disrupt circadian oscillations on soft substrates. We tested this by increasing the baseline expression of BMAL1 and PER/CRY in cells on a soft substrate and observed that the correlation with MRTF nuclear abundance was no longer significant, whereas the correlation with nuclear YAP/TAZ remains significant."

© 2024. Published by The Company of Biologists under the terms of the Creative Commons Attribution License (https://creativecommons.org/licenses/by/4.0/).

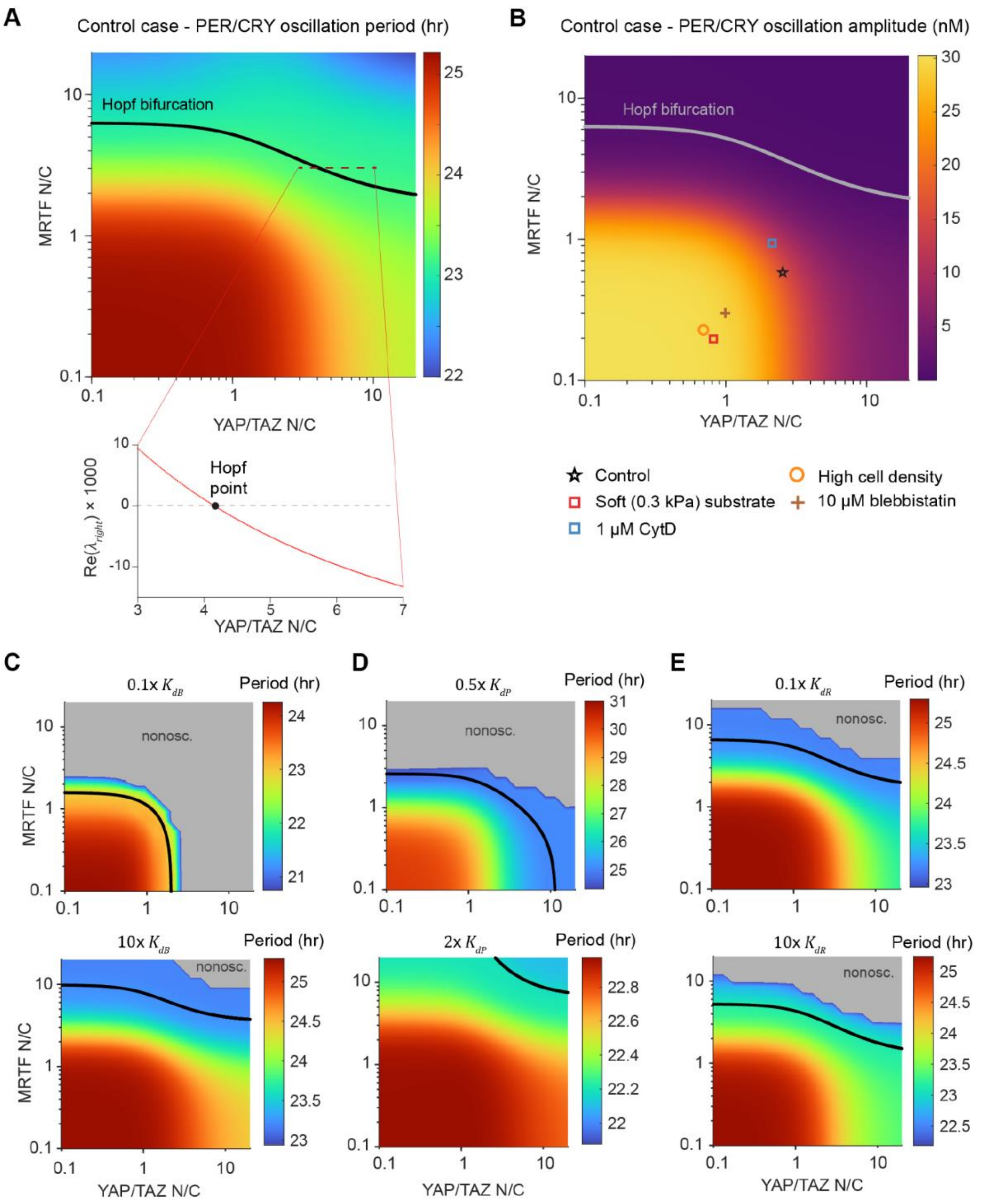

Linear stability analysis for different parameter values. A) Bifurcation diagram for control conditions, with oscillation period and location of the Hopf bifurcation plotted as a function of YAP/TAZ and MRTF nuclear to cytosolic ratios (N/C). The real part of the right-most pair of eigenvalues (λ_{right}) along the line where MRTF N/C is 3 (red dashed line in upper plot). The transition from positive to negative real part of the eigenvalues marks the occurrence of a Hopf bifurcation. B) Oscillation amplitude of PER/CRY over the MRTF-YAP/TAZ phase plane. Individual points correspond to tests run in Figure 5. C-E) Effects of decreasing or increasing K_{dB} (C), K_{dP} (D), or K_{dR} (E) on the location of the Hopf bifurcation and the oscillation period. The grey regions denote nonoscillatory solutions. increasing the baseline expression of BMAL1 and PER/CRY in cells on a soft substrate and observed that the correlation with MRTF nuclear abundance was no longer significant, whereas the correlation with nuclear YAP/TAZ remains significant.”

© 2024. Published by The Company of Biologists under the terms of the Creative Commons Attribution License (https://creativecommons.org/licenses/by/4.0/).

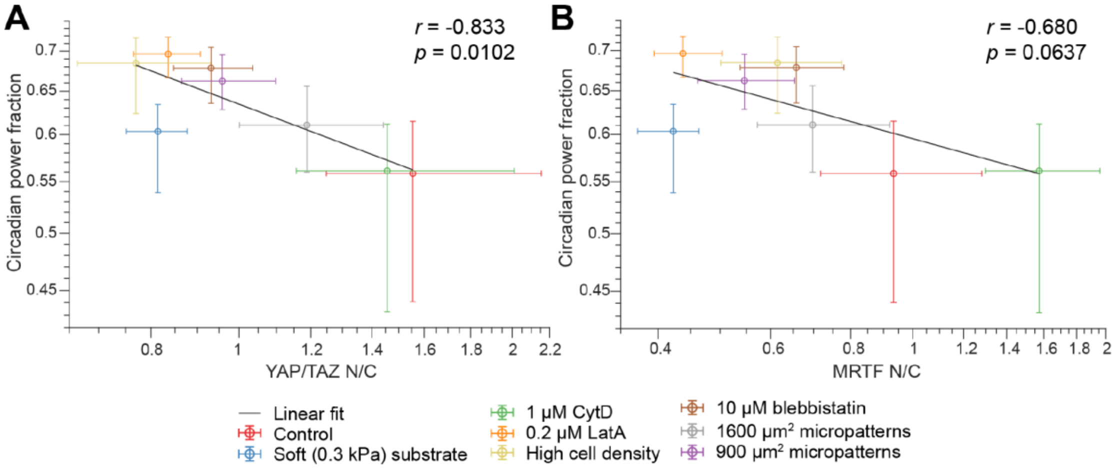

Altered power fraction / transcriptional regulator correlation with increased expression of PER/CRY and BMAL1 on soft substrates. Increasing the baseline expression of PER/CRY and BMAL1 for cells on a soft substrate changes the correlations between circadian power fraction and YAP/TAZ N/C (A) or the MRTF N/C (B). In this case, we added additional baseline expression terms to Equations 9 and 10, each 0.0175 hr^{-1}. Pearson's correlation coefficient (between median YAP/TAZ or MRTF N/C and median circadian power fraction, both on the log scale), *r*, and the *p*-value associated with the null hypothesis, $r = 0$, are included on each graph. Data points denote median and error bars denote the range from the 40th to the 60th percentile for 200 model cells. To generate distinguishable colors for each condition, we used the linspecer tool in MATLAB (Lansey 2023).

16. *". . . model by introducing an equal amount of mutant 5SA-YAP as native YAP/TAZ" And keeping the total amount of YAP constant? (so replacing half of the native by mutant) Or increasing the total amount of YAP by half?*
    Our apologies for the lack of clarity on this point. In these simulations, we kept amount of native YAP/TAZ equivalent to control conditions and added an equivalent amount of 5SA-YAP. Accordingly, the total YAP/TAZ concentration was twice the reference amount; we have clarified this in lines 834-839: "We tested this condition in our model by introducing a new species, 5SA-YAP, which behaved the same as wild-type YAP/TAZ, but had a phosphorylation rate equal to zero. In these tests, the amount of wild-type YAP/TAZ and mutant YAP were each equal to the amount of YAP/TAZ in control cells, resulting in twice the overall YAP/TAZ per cell."
17. *For the mutations, did you also explore other settings, e.g. various opening rate of NPCs and various mutant 5SA-YAP levels (not equal amount)? How does that influence your conclusions?*
    We show in the new Figure S5 that the same trends are followed for different baseline choices of 5SA-YAP expression level and NPC opening rate. This is now noted in lines 892-895: "We ensured the robustness of our results by testing different choices of mutation-related parameters for LMNA and 5SA-YAP, as shown in Figure S5." We also note that these values are not fixed, but vary with log-normal distributions throughout the cell populations.
18. *Typos: -Line 29 - REV-ERBα – also write in full? -that mutated the lamin 300 A was constitutively active → that the mutated lamin A. . . (typo)*
    Thank you for noting these mistakes, these are fixed in the revised version of the manuscript.

© 2024. Published by The Company of Biologists under the terms of the Creative Commons Attribution License (https://creativecommons.org/licenses/by/4.0/).

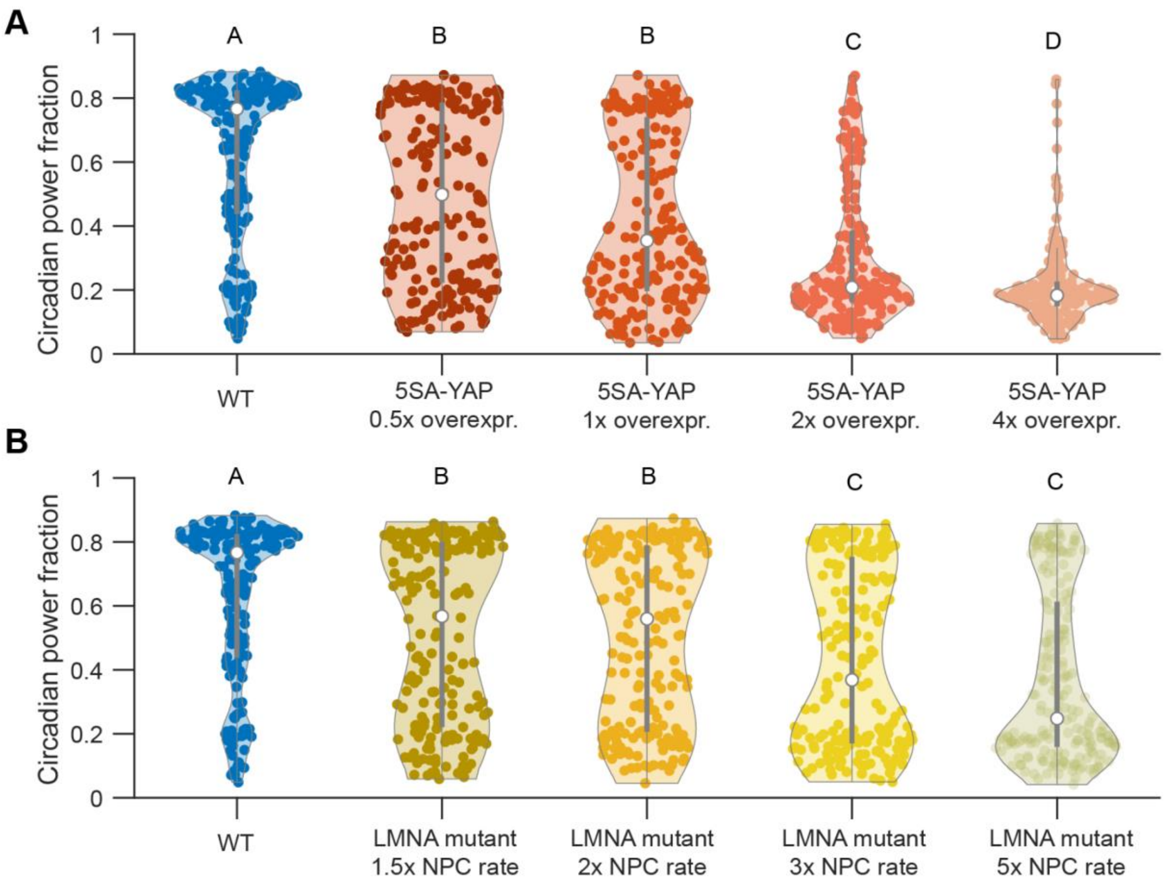

Effects of alternative mutation parameters on model predictions. Circadian power fractions plotted for cells with different overexpression levels of YAP mutant (A) and different changes in NPC opening rate induced by LMNA mutation (B). 5SA-YAP overexpression is controlled by $Koverexpress$ and NPC opening rate is altered by changing K_{fNPC} (Tables 2, 3, 5). Compact letter display is used to denote statistical significance, where groups sharing a letter are statistically similar according to ANOVA followed by Tukey's *post hoc* test with a significance threshold of p= 0.05.

Reviewer 2

Advance Summary and Potential Significance to Field:

This in silico modelling paper by Francis and Rangamani is a very interesting read. It is very well written. It builds on recent experimental observations for mechanical control of cell intrinsic clocks, previous modelling of clocks and mechanical pathways. There are several new directions predicted that can be experimentally validated in future, such as the mechanical rescue of disrupted rhythms due to mutations in YAP or Lamin. The finding that YAP is more dominant than MRTF in regulating clocks is also new and relevant. These findings will shed light on this emerging new model of regulation of circadian clocks by tissue mechanics, which could have implications in cancer, fibrosis or laminopathies. However, there are significant flaws in the modelling parameters, which prevented enthusiasm and must be addressed before further consideration.

We thank the reviewer for their thoughtful feedback. We have noted both the enthusiasm and concerns that the reviewer has raised. We have addressed the concerns and respond to them below.

Reviewer 2 Comments for the Author:

19. *The use of nuclear BMAL1 to measure clock dynamics is unfortunate. It has been shown by quantitative imaging and math models that BMAL1 proteins hardly oscillate in the nucleus with a very small amplitude, which is very different from what is being proposed in the modelling here (e.g. Fig.4 and Fig.1B/C). It is well known that the negative arm of the clock is the more rhythmic arm. Please see PMID:*

© 2024. Published by The Company of Biologists under the terms of the Creative Commons Attribution License (https://creativecommons.org/licenses/by/4.0/).

19549830; PMID: 32352975

Thank you for this helpful feedback; we have taken it into account in a modified version of our computational model. Our original model relied heavily on oscillations in BMAL1 driving oscillations in PER/CRY, but we agree this is not consistent with previous literature. Accordingly, we made the following key changes to our model:

- We assume the delay-regulated expression of BMAL1 (K_{eB0}) is small in comparison to delay- regulated terms for other variables (one order of magnitude smaller in baseline parameters).
- We add an additional PER/CRY delay-regulated expression term ($K_{eP,P}$). Originally, the indirect self-inhibition of PER/CRY species was modeled by the degradation of the BMAL1-CLOCK- PER/CRY complex in the nucleus, leading to downstream decreases in PER/CRY expression. However, upon enforcing smaller BMAL1 oscillations, this formulation of PER/CRY self inhibition does not drive oscillations. Accordingly, we introduced a direct negative feedback term with time delay, as in (Sriram et al. 2006), which produces oscillations even for constant BMAL1.
- Finally, we add an additional variable, R, to represent REV-ERBα. This allows us to more realistically represent negative feedback on expression of BMAL1. In line with previous models, we assume it is positively regulated by BMAL1 and negatively regulated by PER/CRY (Mirsky et al. 2009; Sriram et al. 2006). We also allow for mechanotransduction-mediated changes in REV-ERBα expression, but these have a small impact on overall oscillations, as REV-ERBα only mediates small oscillations in BMAL1.

We hope the reviewer will agree that this new version of our model aligns better with the current understanding of the mammalian circadian clock and still preserves our attempt to create a minimal model of this system. The new model behavior is summarized in our updated Figure 1 as shown below.

20. *This paper has not taken into account cell-type dependent responses of clocks to mechanical cues, which as been reported before at least for epithelial cells and fibroblasts. The paper was cited but not properly discussed, nor modelled.*

We agree that this is a very important discussion point not properly addressed in our original paper. Accordingly, we have added a paragraph to the discussion comparing our results here to the relevant cell-type dependent results in (Williams et al. 2018). An important difference from our study comes from the fact that our model is calibrated to cells on 2D substrates, whereas the stiffness-dependent behavior in Williams et al. (Williams et al. 2018) was established in 3D. The apparent discrepancy between the fibroblast behavior in Xiong et al. (Xiong et al. 2022) and that in Williams et al. may be accounted for

© 2024. Published by The Company of Biologists under the terms of the Creative Commons Attribution License (https://creativecommons.org/licenses/by/4.0/).

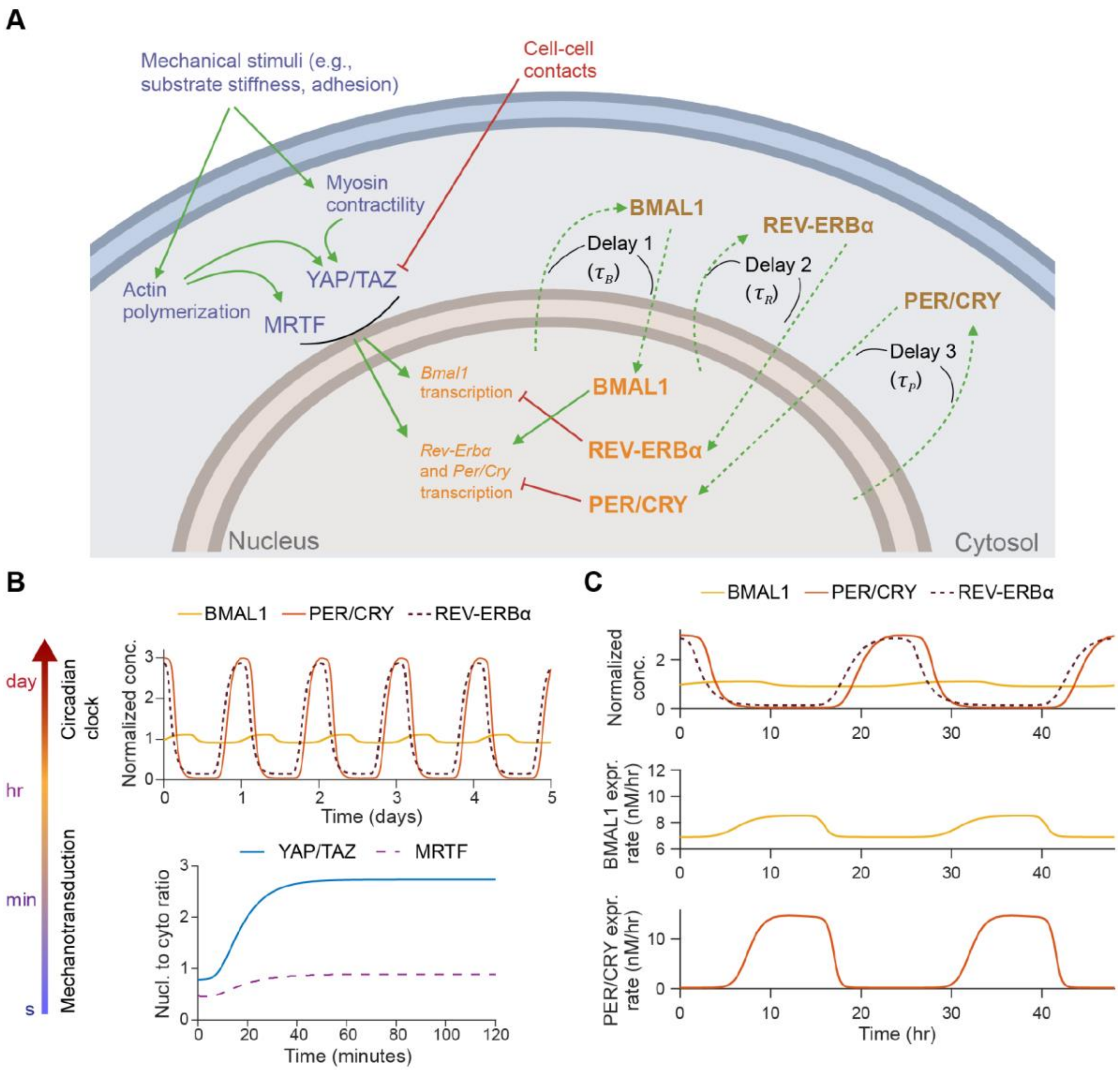

A coupled model of mechanotransduction and the circadian clock. A) Schematic of pathways for YAP/TAZ and MRTF-mediated mechanotransduction and the mammalian circadian clock. Mechanical stimuli such as substrate stiffness and cell-substrate adhesion induce changes in cytoskeletal activity that lead to nuclear translocation of YAP/TAZ and MRTF. Cell-cell contacts also inhibit YAP/TAZ activation via a LATS-dependent pathway. Nuclear YAP/TAZ and MRTF are posited to induce changes in the expression of BMAL1, PER, CRY, and REV-ERBα proteins, altering circadian oscillations. In parallel, increases in nuclear BMAL1 enhance transcription of *Per*, *Cry*, and *Rev-ERb*α, while increases in nuclear REV-ERBα inhibit transcription of *Bmal1* and increases in nuclear PER/CRY inhibit transcription of *Per*, *Cry*, and *Rev-ERb*α. Dashed lines indicate delays associated with transcription, translation, post-translational modifications, and nuclear translocation of BMAL1, PER/CRY, and REV-ERBα. The schematic was created with Biorender.com. B) Separation of timescales from mechanotransduction pathways to circadian oscillations. Events downstream of mechanical stimuli lead to changes in YAP/TAZ and MRTF nuclear concentrations within tens of minutes (lower plot), whereas changes in the concentration of circadian proteins are extended over hours to days (upper plot). C) Simulated dynamics of single-cell circadian oscillations. BMAL1, PER/CRY, and REV-ERBα all oscillate with periods close to 1 day. Rates of expression of BMAL1 and PER/CRY are depicted in lower plots.

either by cell-line/organ-specific differences in fibroblasts or (perhaps more likely) by the well-established differences between cell behavior in 2D vs. 3D. Furthermore, we note that stiffness is only one aspect of material properties; ECM differs in other important ways from hydrogels. We note that we are currently considering these different aspects in detailed models but parsing out all of these important factors is a follow-up study. The

© 2024. Published by The Company of Biologists under the terms of the Creative Commons Attribution License (https://creativecommons.org/licenses/by/4.0/).

paragraph added to discussion reads:
"The overall trend of weaker circadian oscillations for increased mechanical activation agrees well with previous measurements in epithelial cells, but the same study measured the opposite trend in mouse fibroblasts (Williams et al. 2018). This apparently disagrees with the trend observed in mouse fibroblasts by Xiong et al. (Xiong et al. 2022). However, we note that the study by Williams et al. only assessed cell responses to softer hydrogels in 3D culture (Williams et al. 2018), whereas Xiong et al. made all measurements on 2D substrates (Xiong et al. 2022). Aside from tissue- or organism-specific differences in fibroblast behavior, the difference in observations could be attributed to the well-established differences between cell behavior in 2D vs. 3D culture (Baker and Chen 2012; Duval et al. 2017). This could be investigated in future modeling work examining the effects of different spatial stimuli on cell mechanotransduction (Scott et al. 2021)."

21. *It is unclear how cells on glass culture, which the authors mentioned to be extremely stiff (10 GPa), can be used for amplitude normalisation in Figure 2.*
We have responded to this comment as we have understood it. If we have misunderstood the reviewer comment, then we apologize and ask for further clarification. In our model, while oscillations are smaller in magnitude on stiff substrates, they are still robust (the system is not past the Hopf bifurcation). This agrees well with experiments, where most cells still exhibit oscillations in circadian proteins on tissue culture plastic (Xiong et al. 2022). Accordingly, there is no issue using this condition for amplitude normalization, and it allows us to leverage our model predictions against experimental measurements.
22. *There is a misunderstanding of the term entrainment, which is different from clock resetting, synchronisation, or modulation. To qualify for an entrainment time cue, the signal got to be rhythmic which then entrain the phase of the oscillation. As such, the word "entrainment cue" in the title and throughout the paper should be replaced, for example, with "Regulator" or "modulator".*
Thank you for this important clarification, we have replaced "entrainment" throughout the text and in the title, as we agree we have not demonstrated entrainment in our current tests. The new title reads "Computational modeling establishes mechanotransduction as a potent modulator of the mammalian circadian clock."

References

Abenza, Juan F. et al. (Sept. 4, 2023). "Mechanical Control of the Mammalian Circadian Clock via YAP/TAZ and TEAD". In: *Journal of Cell Biology* 222.9, e202209120. issn: 0021-9525, 1540-8140.

Baker, Brendon M. and Christopher S. Chen (July 1, 2012). "Deconstructing the Third Dimension – How 3D Culture Microenvironments Alter Cellular Cues". In: *Journal of Cell Science* 125.13, pp. 3015–3024. issn: 0021-9533. pmid: 22797912.

Cenni, V (Mar. 1, 2005). "Lamin A N-terminal Phosphorylation Is Associated with Myoblast Activation: Impairment in Emery-Dreifuss Muscular Dystrophy". In: *Journal of Medical Genetics* 42.3, pp. 214–220. issn: 1468-6244.

Dutta, Subarna et al. (Sept. 14, 2018). "Skeletal Muscle Dystrophy Mutant of Lamin A Alters the Structure and Dynamics of the Ig Fold Domain". In: *Scientific Reports* 8.1, p. 13793. issn: 2045-2322.

Duval, Kayla et al. (July 2017). "Modeling Physiological Events in 2D vs. 3D Cell Culture". In: *Physiology* 32.4, pp. 266–277. issn: 1548-9213. pmid: 28615311.

Forger, Daniel B. and Charles S. Peskin (Dec. 9, 2003). "A Detailed Predictive Model of the Mammalian Circadian Clock". In: *Proceedings of the National Academy of Sciences of the United States of America* 100.25, pp. 14806–14811. issn: 0027-8424. pmid: 14657377.

Koch, Arthur L. (Nov. 1, 1966). "The Logarithm in Biology 1. Mechanisms Generating the Log-Normal Distribution Exactly". In: *Journal of Theoretical Biology* 12.2, pp. 276–290. issn: 0022-5193.

Lansey, Jonathan (2023). *Beautiful and Distinguishable Line Colors + Colormap.*

Leloup, Jean-Christophe and Albert Goldbeter (June 10, 2003). "Toward a Detailed Computational Model for the Mammalian Circadian Clock". In: *Proceedings of the National Academy of Sciences* 100.12, pp. 7051– 7056. issn: 0027-8424, 1091-6490.

Lema, Mart´ın A., Diego A. Golombek, and Juli´an Echave (June 2000). "Delay Model of the Circadian Pace- maker". In: *Journal of Theoretical Biology* 204.4, pp. 565–573. issn: 00225193.

© 2024. Published by The Company of Biologists under the terms of the Creative Commons Attribution License (https://creativecommons.org/licenses/by/4.0/).

Mirsky, Henry P., Andrew C. Liu, David K. Welsh, Steve A. Kay, and Francis J. Doyle (July 7, 2009). “A Model of the Cell-Autonomous Mammalian Circadian Clock”. In: *Proceedings of the National Academy of Sciences of the United States of America* 106.27, pp. 11107–11112. issn: 0027-8424. pmid: 19549830.

Mitsuhashi, Hiroaki et al. (Nov. 15, 2010). “Specific Phosphorylation of Ser458 of A-type Lamins in *LMNA* -Associated Myopathy Patients”. In: *Journal of Cell Science* 123.22, pp. 3893–3900. issn: 1477-9137, 0021-9533.

Ni, Haibo, Stefano Morotti, and Eleonora Grandi (July 20, 2018). “A Heart for Diversity: Simulating Variability in Cardiac Arrhythmia Research”. In: *Frontiers in Physiology* 9, p. 958. issn: 1664-042X.

Owens, Daniel J. et al. (Mar. 27, 2020). “Lamin Mutations Cause Increased YAP Nuclear Entry in Muscle Stem Cells”. In: *Cells* 9.4, p. 816. issn: 2073-4409.

Scott, Kiersten Elizabeth, Stephanie I. Fraley, and Padmini Rangamani (May 18, 2021). “A Spatial Model of YAP/TAZ Signaling Reveals How Stiffness, Dimensionality, and Shape Contribute to Emergent Outcomes”. In: *Proceedings of the National Academy of Sciences* 118.20, e2021571118. issn: 0027-8424, 1091-6490.

Smolen, Paul, Douglas A. Baxter, and John H. Byrne (Sept. 1, 2001). “Modeling Circadian Oscillations with Interlocking Positive and Negative Feedback Loops”. In: *The Journal of Neuroscience* 21.17, pp. 6644– 6656. issn: 0270-6474, 1529-2401.

Sriram, K., Gilles Bernot, and Fran¸cois K´ep`es (2006). “Discrete Delay Model for the Mammalian Circadian Clock”. In: *Complexus* 3.4, pp. 185–199. issn: 1424-8492, 1424-8506.

Swift, Joe et al. (Aug. 30, 2013). “Nuclear Lamin-A Scales with Tissue Stiffness and Enhances Matrix-Directed Differentiation”. In: *Science* 341.6149, p. 1240104. issn: 0036-8075, 1095-9203.

Torvaldson, Elin, Vitaly Kochin, and John E Eriksson (May 4, 2015). “Phosphorylation of Lamins Determine Their Structural Properties and Signaling Functions”. In: *Nucleus* 6.3, pp. 166–171. issn: 1949-1034, 1949-1042.

Williams, Jack et al. (Jan. 1, 2018). “Epithelial and Stromal Circadian Clocks Are Inversely Regulated by Their Mechano-Matrix Environment”. In: *Journal of Cell Science*, jcs.208223. issn: 1477-9137, 0021-9533. Xiong, Xuekai et al. (Oct. 1, 2022). “The Actin Cytoskeleton-MRTF/SRF Cascade Transduces Cellular Physical Niche Cues to Entrain the Circadian Clock”. In: *Journal of Cell Science* 135.19, jcs260094. issn:0021-9533, 1477-9137.

Zhang, C-L. and F-A. Popp (July 1, 1994). “Log-Normal Distribution of Physiological Parameters and the Coherence of Biological Systems”. In: *Medical Hypotheses* 43.1, pp. 11–16. issn: 0306-9877.

Second decision letter

MS ID#: JOCES/2023/261782

MS TITLE: Computational modeling establishes mechanotransduction as a potent modulator of for the mammalian circadian clock

AUTHORS: Emmet A Francis and Padmini Rangamani

Thank you for your patience. We have just received the final review of your revision and I am happy to tell you that your manuscript has been accepted for publication in Journal of Cell Science, pending standard publication integrity checks.

Reviewer 1

Advance summary and potential significance to field

In this study the authors coupled a mechanoregulatory model to a model of the circadian clock. They show that YAP/TAZ and MRTF nuclear accumulation influences the amplitude and frequency

© 2024. Published by The Company of Biologists under the terms of the Creative Commons Attribution License (https://creativecommons.org/licenses/by/4.0/).

of circadian genes/proteins, corresponding to experimental literature. They also show that YAP/TAZ nuclear accumulation seems to play a more influential role than MRTF (in baseline parameter settings, in the revised model) and provide testable predictions to rescue the circadian rhythm in cells with particular mutations.

Comments for the author

All my comments have been carefully addressed, I congratulate the authors on this great work.

Reviewer 2

Advance summary and potential significance to field

This modelling paper is a significant advance to the field of mechano-regulation of cellular circadian clocks. As such, it will facilitate our understanding of how cell intrinsic timers adapt to their microenvironment.

Comments for the author

The authors have carefully addressed my comments. The revision of statements on BMAL1 and the addition of REV-ERB will significantly strengthen the value of the model. The entrainment reference has been removed from abstract and in the text.

Regarding the point below {21. It is unclear how cells on glass culture, which the authors mentioned to be extremely stiff (10 GPa), can be used for amplitude normalisation in Figure 2}, what I meant was that cells cultured on glass had an extremely high stiffness which is nowhere near the physiological conditions.
While stiffness of cells on plastics, although still not physiological, is in-between. I wondered whether cells on plastics is a better normalisation condition in this case. But it is a relatively minor point so I leave it with the authors to consider.

I have no further questions.

© 2024. Published by The Company of Biologists under the terms of the Creative Commons Attribution License (https://creativecommons.org/licenses/by/4.0/).
